# Intelligent Hydrogel-Assisted Hepatocellular Carcinoma Therapy

**DOI:** 10.34133/research.0477

**Published:** 2024-10-14

**Authors:** Zixiang Tang, Lin Deng, Jing Zhang, Tao Jiang, Honglin Xiang, Yanyang Chen, Huzhe Liu, Zhengwei Cai, Wenguo Cui, Yongfu Xiong

**Affiliations:** ^1^ Department of Hepatobiliary Surgery, Academician (Expert) Workstation, Sichuan Digestive System Disease Clinical Medical Research Center, Affiliated Hospital of North Sichuan Medical College, Nanchong 637000, P. R. China.; ^2^ Department of Clinical Medicine, North Sichuan Medical College, Nanchong 637000, P. R. China.; ^3^ Department of Gastroenterology, Affiliated Hospital of North Sichuan Medical College, Nanchong 637000, P. R. China.; ^4^ Department of Orthopaedics, Shanghai Key Laboratory for Prevention and Treatment of Bone and Joint Diseases, Shanghai Institute of Traumatology and Orthopaedics, Ruijin Hospital, Shanghai Jiao Tong University School of Medicine, Shanghai 200025, P. R. China.

## Abstract

Given the high malignancy of liver cancer and the liver’s unique role in immune and metabolic regulation, current treatments have limited efficacy, resulting in a poor prognosis. Hydrogels, soft 3-dimensional network materials comprising numerous hydrophilic monomers, have considerable potential as intelligent drug delivery systems for liver cancer treatment. The advantages of hydrogels include their versatile delivery modalities, precision targeting, intelligent stimulus response, controlled drug release, high drug loading capacity, excellent slow-release capabilities, and substantial potential as carriers of bioactive molecules. This review presents an in-depth examination of hydrogel-assisted advanced therapies for hepatocellular carcinoma, encompassing small-molecule drug therapy, immunotherapy, gene therapy, and the utilization of other biologics. Furthermore, it examines the integration of hydrogels with conventional liver cancer therapies, including radiation, interventional therapy, and ultrasound. This review provides a comprehensive overview of the numerous advantages of hydrogels and their potential to enhance therapeutic efficacy, targeting, and drug delivery safety. In conclusion, this review addresses the clinical implementation of hydrogels in liver cancer therapy and future challenges and design principles for hydrogel-based systems, and proposes novel research directions and strategies.

## Introduction

Hepatocellular carcinoma (HCC), which accounts for 80 to 90% of primary liver cancer cases, represents the seventh most prevalent malignancy worldwide, accounting for 4.7% of new cancer diagnoses and is the third leading cause of cancer deaths, at 8.3% of cancer-related mortalities. The close correlation between incidence rates and mortality, with 83,000 annual fatalities, underscores the grave prognosis associated with this disease [1]. Geographical and ethnic variations in HCC incidence are substantially influenced by the prevalence and timing of exposure to key risk factors. The major recognized risk factors contributing to the development of HCC include infection with the hepatitis B virus (HBV) or the hepatitis C virus (HCV), alcohol consumption, nonalcoholic steatohepatitis (NASH), and exposure to aflatoxin B1 [2]. The global distribution of HCC risk factors demonstrates notable variation, with HBV being more prevalent in China, HCV in Japan, and nonalcoholic fatty liver disease (NAFLD), NASH, and alcohol consumption in Europe and North America. The probability of HCC development is influenced by a multitude of factors, including demographic characteristics (such as age, sex, and ethnicity), the severity and progression of the underlying condition (including the stage of fibrosis and inflammatory activity, as well as the impact of treatment), metabolic conditions (such as diabetes mellitus and obesity), and lifestyle choices (notably alcohol consumption and smoking) [3].

The liver’s distinctive dual blood supply, delivered by both the hepatic artery and the portal vein, renders it particularly well suited for transarterial therapies. Liver tumors primarily rely on arterial blood flow for sustenance, in contrast to the rest of the liver, which is nourished by the portal vein. This distinctive vascular configuration allows for the administration of intra-arterial therapy (IAT) via catheters [4], which actively targets drug therapy to the tumor while preserving the remaining liver tissue. The tumor microenvironment (TME) is composed of a heterogeneous collection of cells and components that collectively facilitate tumor progression, invasion, and metastasis while impeding the response to therapy [5]. HCC is characterized by a highly immunosuppressive microenvironment that effectively resists both drug therapy and immune attacks [6], and it engenders a complex milieu through multiple metabolic responses that increase tumor invasiveness and metastatic potential [7] and exacerbate resistance to treatment. The current clinical treatments for HCC include surgery, interventional therapy, radiotherapy, chemotherapy, and emerging biotherapies [8]. Conventional surgical procedures have a high risk of recurrence and cannot confer long-term immunity against cancer [9]. Interventional therapies, including ablation and IAT, might not completely eradicate the tumor and can fail to prevent the recurrence or metastasis of HCC [10]. Radiotherapy and chemotherapy, while widely used as nonsurgical treatments for HCC, exhibit limited efficacy, particularly in advanced tumors [11]. In summary, these therapies encounter challenges due to the distinctive physiology of the liver and the complex nature of the TME, which increase the complexity of HCC and create an imperative need for innovative therapeutic approaches.

**Fig. 1.**
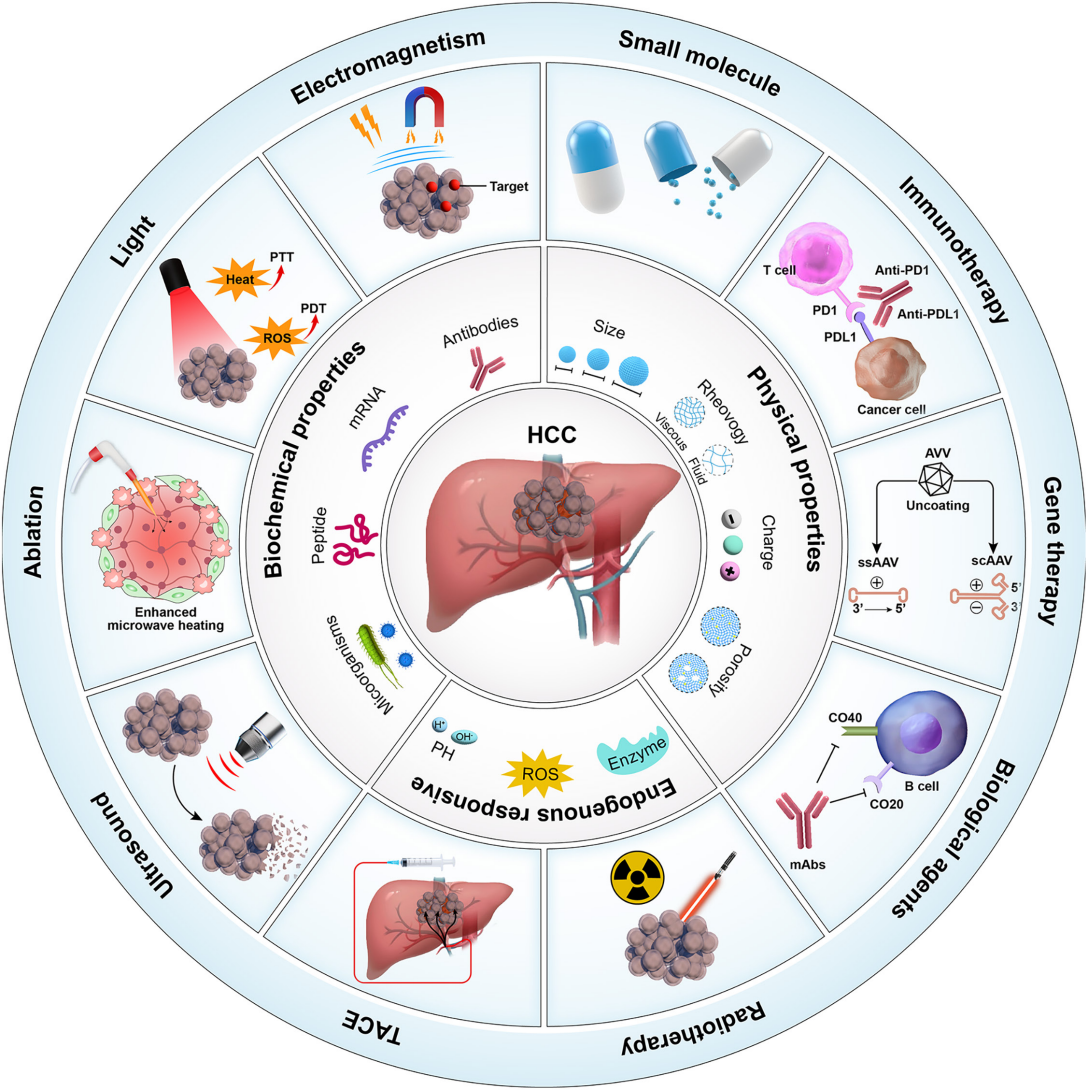
Comprehensive therapeutic strategies for HCC: the application of intelligent hydrogels.

Rapid advances in drug delivery technologies, including hydrogels, in situ gelling systems, liposomes, nanoparticles, and micelles, has demonstrated considerable potential in enhancing tumor prognosis. Among these technologies, hydrogels have received considerable attention as vital drug delivery system [12]. First, hydrogels can achieve precise drug targeting through the recognition of liver cancer-specific markers or by exploiting unique physicochemical properties within the TME, such as specific enzyme expression [13]. Second, hydrogels, especially those that are responsive to external stimuli, are highly effective in controlled release. Stimuli-responsive hydrogels can regulate drug release in response to specific stimuli, such as pH or interactions with reactive oxygen species (ROS), in the TME of HCC, thereby achieving precise controlled release [14]. Third, the distinctive 3-dimensional (3D) network and highly porous nature of hydrogels endow them with a large drug loading capacity and significant slow-release capability. The modifiable structure allows for precise, sustained drug release with stable rates [12]. Last, the physicochemical characteristics of hydrogels render them ideally suited for encapsulating a diverse array of biologically active molecules [15], such as RNA, proteins, or peptides; these molecules are particularly vulnerable to the harsh conditions of the digestive tract, which challenge their stability and efficacy in traditional drug delivery systems. The acidic pH of the stomach, combined with the near-neutral pH of the small intestine, can lead to drug degradation and denaturation. Additionally, digestive enzymes like pepsin, trypsin, and nucleases contribute to enzymatic breakdown, while the mucosal barriers and efflux transporters further limit drug absorption and bioavailability [16] (Fig. 1). Consequently, hydrogels not only offer new possibilities for liver cancer treatment but also provides a foundation for future research in cancer treatment.

This review provides a comprehensive examination of the potential of hydrogels as an innovative material for the treatment of HCC, focusing on localized targeting within the liver and an analysis of the specific challenges of the TME in HCC. This paper provides a detailed account of the versatility and efficacy of hydrogels as state-of-the-art biomaterials for targeted drug delivery, immunotherapy, gene therapy, and the delivery of other biologics. Moreover, we highlight the significant potential and wide-ranging advantages of employing hydrogels in combination with traditional therapeutic modalities, including radiation, interventional, and ultrasound therapies. This approach is intended to optimize therapeutic efficacy and increase the precision and safety of drug delivery. Given their efficacy and versatility, hydrogels are particularly well-suited as drug delivery platforms for the treatment of HCC. The unique biological characteristics of HCC and the role of hydrogels in enhancing the precision and efficacy of treatment make them an optimal selection. As related technologies continue to evolve, the application of hydrogel systems in cancer therapy, particularly liver cancer therapy, holds considerable promise.

## Histologic Anatomy and Microenvironment of HCC

As a global health challenge, HCC presents a distinctive set of physiological and microenvironmental complexities that demand more sophisticated therapeutic strategies; therefore, it is imperative to conduct rigorous research and develop innovative new strategies. This section will examine the physiological characteristics of the liver and the impact of the HCC TME on treatment, and it will also analyze how these aspects can be leveraged to foster the development of innovative therapeutic strategies.

### Tissue anatomy of HCC

**Fig. 2.**
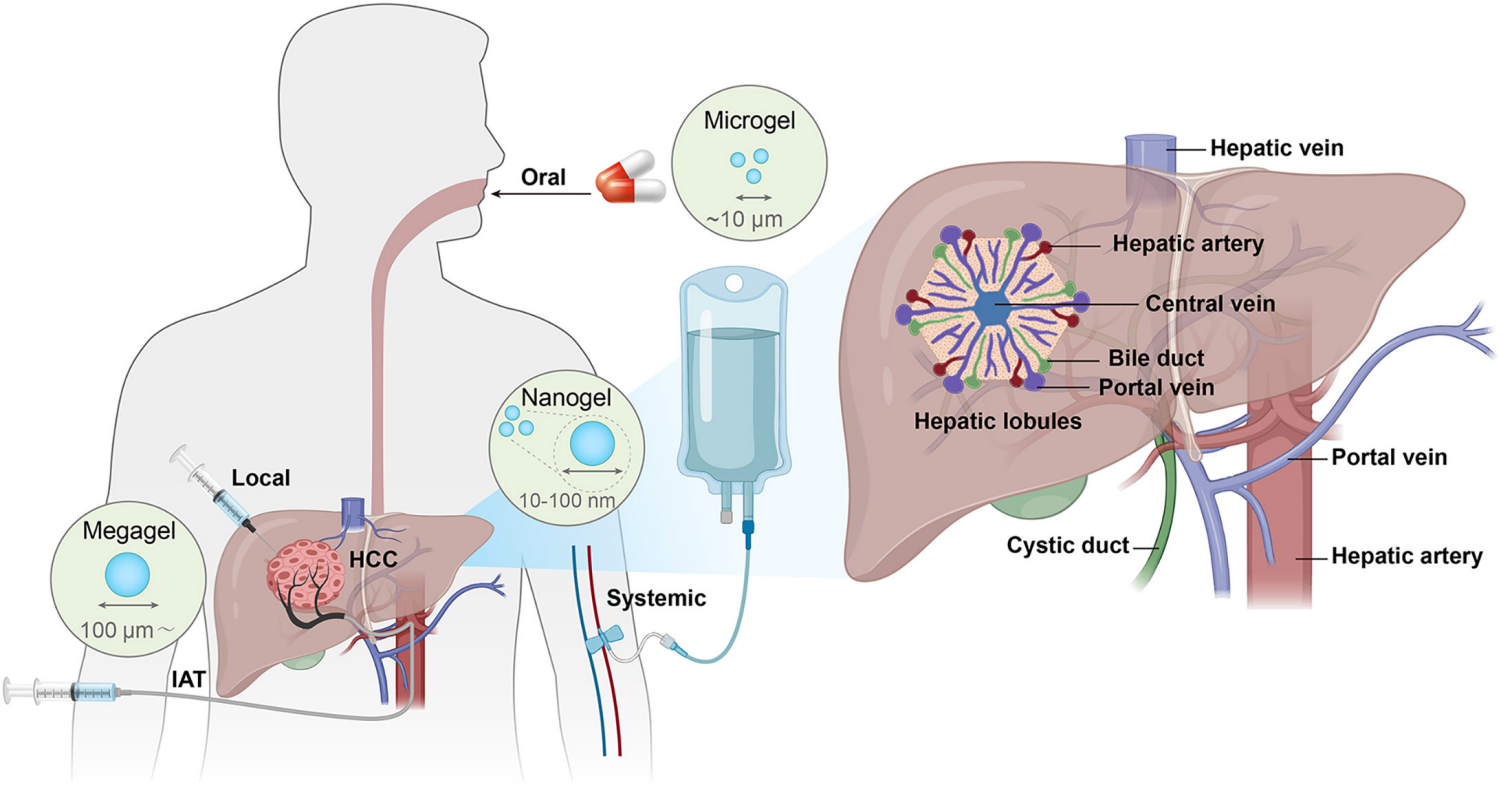
Hydrogel-assisted HCC administration and specialized anatomy of the liver.

The liver’s distinctive anatomical characteristics are attributable to dual blood supply system, which encompasses both arterial and venous sources. This unique configuration enables the targeted application of IAT, as illustrated in Fig. 2. This arrangement allows a blend of arterial and venous blood to nourish liver structures and cells. Oxygenated blood is delivered through the hepatic artery, which constitutes a smaller portion of the liver’s blood supply, while the portal vein, carrying nutrient-rich but pathogen-containing blood, furnishes the bulk of the liver’s circulation [17]. Liver tumors are primarily supplied by the hepatic artery, whereas the majority of the normal liver parenchyma is nourished by the portal vein. Infusing cancer therapies through the hepatic artery facilitates selective drug delivery to tumors, sparing normal liver tissue and minimizing extrahepatic exposure, thereby reducing systemic side effects [18]. Furthermore, the low-pressure blood from the hepatic portal vein enters the hepatic sinusoids through extensive branching, which is further slowed down by the wide and irregular structure of the sinusoids. This anatomical structure supports the hypothesis that the liver plays a role in the intravascular immune response [19]. Molecules derived from microbes, such as pathogen-associated molecular patterns (PAMPs), and substances needing clearance flow into the liver’s honeycomb-like hepatic sinusoids at lowered velocities and pressures [17]. This decreased flow rate increases the interaction of the blood with Kupffer cells (KCs) and liver sinusoidal endothelial cells (LSECs), both of which are essential for filtering the blood to eliminate pathogens and undesirable molecules. This distinctive liver architecture provides a physiological basis for treatments targeting liver tumors.

### TME in HCC

**Fig. 3.**
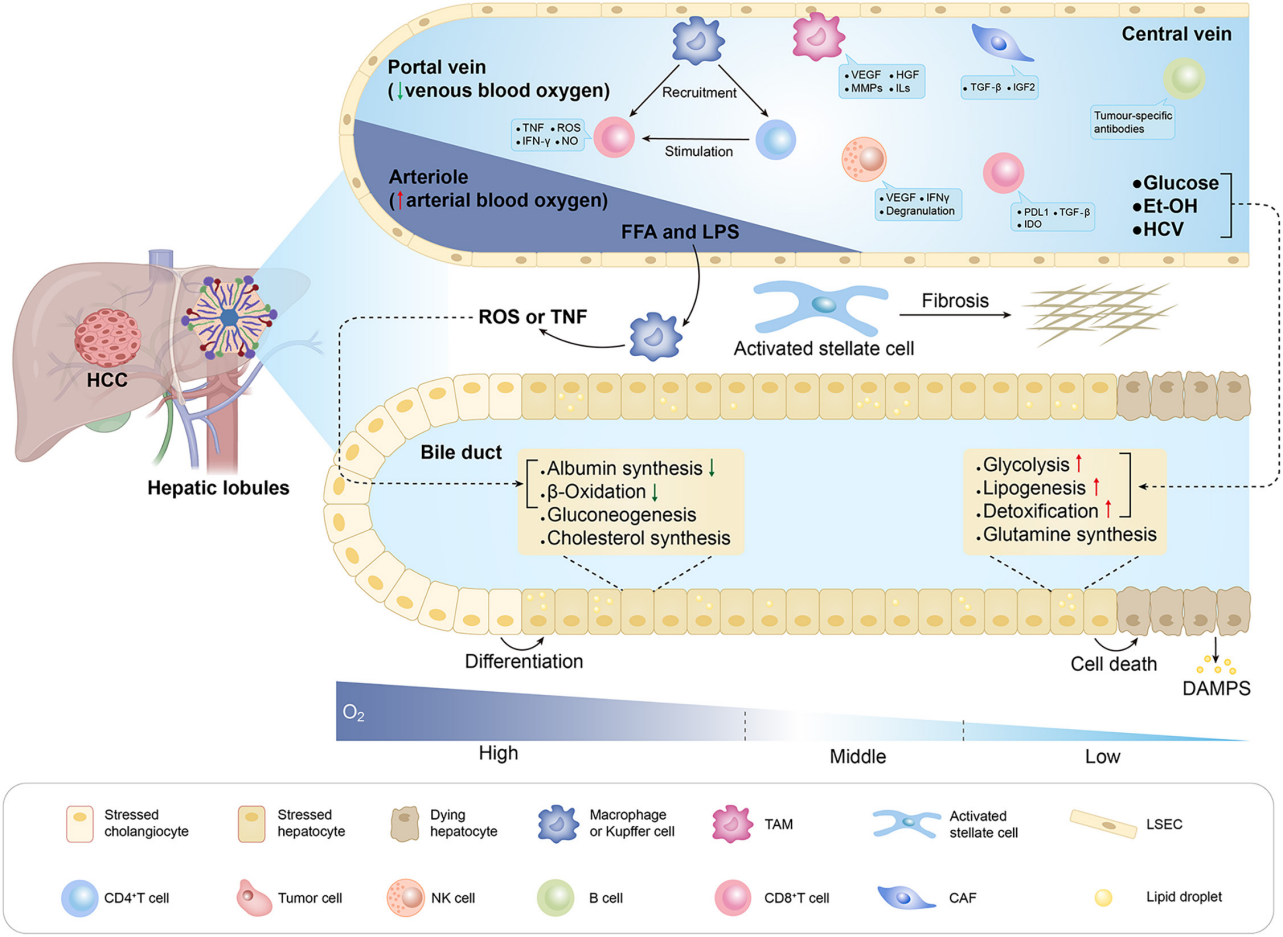
Immune microenvironment and metabolic microenvironment in HCC.

The TME of HCC is both dynamic and intricate, comprising a multitude of components, including cancer cells, stromal cells, blood vessels, nerve fibers, the extracellular matrix (ECM), and associated decellularized elements. The TME can be categorized into various niches: immune, metabolic, hypoxic, acidic, neurological, and mechanical. Each of these niches exerts a profound influence on cancer progression. The immune and metabolic microenvironments are key factors in determining the outcome of HCC treatment and greatly limit the effectiveness of current therapies (Fig. 3) [20]. The TME is a critical regulator of hepatocarcinogenesis and the progression of liver cancer and serves as a source for identifying potential therapeutic targets [21]. Therefore, reconstructing the TME can significantly influence the malignant behavior of tumor cells, and a detailed analysis of the immune and metabolic microenvironments within HCC is crucial because of their therapeutic implications.

#### The immune environment of HCC

The liver is typically an immune-privileged site with relatively high resistance to immune reactions [22]. As the gatekeeper organ for metabolism, the liver constantly encounters pathogens from the gut, microbe-associated molecular patterns, Toll-like receptor agonists (e.g., TLR4 and TLR9), and various metabolites. Consequently, the liver maintains a default state of immune tolerance, characterized by immunosuppressive polarization that undermines effective T cell-mediated antigenic responses. This state is maintained by a population of resident hepatocytes, including KCs, hepatic stellate cells (HSCs), dendritic cells (DCs), regulatory T cells (T_regs_), and hepatic sinusoidal endothelial cells (LSECs) [23]. However, in the HCC tumor immune microenvironment (TIME), the predominant immunosuppressive cells include tissue-resident macrophages (primarily KCs), myeloid-derived suppressor cells (MDSCs), T_regs_, and monocyte-derived macrophages [24]. These cells facilitate immune evasion and limit the effectiveness of cancer immunotherapy. KCs respond to antigenic, damage-associated, or microbe-derived signals by secreting IL-10 and other immunosuppressive cytokines, and they express the immune checkpoint ligand PD-L1, which significantly hampers the immune system’s capacity to identify and eliminate tumor cells. Additionally, MDSCs promote the induction of T_regs_ and M2 tumor-associated macrophages (M2-TAMs) and inhibit immune effector cells, such as CD8^+^ T cells, DCs, and natural killer (NK) cells, further undermining the immune response against tumors [25]; T_regs_ perform their suppressive role by releasing inhibitory cytokines, notably transforming growth factor-β (TGF-β) and interleukin-10 (IL-10). These substances facilitate the cytolysis and “metabolic destruction” of effector T cells and impede the maturation of DCs [26]. Moreover, the majority of HCC tumors arise within the setting of chronic liver diseases, including cirrhosis and hepatic fibrosis [3], a condition that up-regulates inflammatory signaling. This dysregulation disrupts homeostasis in association with necroinflammation, creating an inflammatory imbalance that severely impacts the TIME, fosters cancerous cell growth, and establishes a safe ecological niche to counteract immune system activation [27]. Therefore, reconstructing the TME can significantly influence the malignant behavior of tumor cells, and a detailed analysis of the immune and metabolic microenvironments within HCC is crucial because of their therapeutic implications [28].

#### Metabolic environment of HCC

The liver, a vital metabolic organ, harbors a diverse array of metabolic enzymes, regulates numerous pathways, and is instrumental in sustaining systemic homeostasis [23]. During the development of a tumor, there are notable alterations in the metabolic pathways, which result in the formation of a unique metabolic microenvironment. Tumor cells deplete their blood supply to meet proliferative demands, leading to chronic or transient hypoxic states and triggering a hypoxia–reoxygenation cycle. This process increases the intracellular concentration of ROS and thus triggers the activation of a cascade of antioxidant enzymes, including superoxide dismutase (SOD), glutathione peroxidase (GPx), and catalase (CAT). It also stimulates the synthesis of antioxidants, such as reduced glutathione (GSH), to counteract oxidative stress. These metabolic changes lead to DNA damage and promote the generation of mutations, which are key drivers of cancer development and progression [29]. Additionally, ROS can act as signaling molecules, activating multiple intracellular pathways, such as the nuclear factor κB (NF-κB) and hypoxia-inducible factor-1α (HIF-1α) pathways, to facilitate the proliferation and survival of cancer cells [30]. Tumor and stromal cells within the TME generate elevated ROS levels, influencing cancer cell growth [31]. Furthermore, within the TME, tumor cells and their stromal counterparts undergo transcriptional reprogramming, leading to a marked increase in the expression of ECM-degrading enzymes, such as matrix metalloproteinases (MMPs). These enzymes play crucial roles in ECM remodeling and destruction by dismantling components such as collagen and fibronectin [32]. Additionally, tumor cells release growth factors that recruit fibroblasts to the TME, where they are transformed into cancer-associated fibroblasts (CAFs), which are characterized by enhanced proliferative capabilities and a tendency to contribute to ECM accumulation. Consequently, the hardened ECM accelerates tumor cell growth, and this interaction creates a positive feedback loop that promotes cancer development [33]. Furthermore, under the Warburg effect, cancer cells undergo anaerobic glycolysis and produce large amounts of lactic acid, thereby lowering the pH of the metabolic microenvironment to between 6.5 and 6.9 [34]. These changes in the metabolic microenvironment observed in HCC limit the efficacy of existing therapies, thereby necessitating the design of more precise and effective therapeutic strategies.

### Current clinical treatment strategies for HCC

The Barcelona Clinical Liver Cancer (BCLC) staging system classifies HCC into 5 distinct clinical stages: very early (BCLC 0), early (BCLC A), intermediate (BCLC B), advanced (BCLC C), and terminal (BCLC D) [3]. For patients diagnosed with early-stage HCC, the preferred treatment options include resection, transplantation, and local ablation. Surgical resection, encompassing hepatectomy and liver transplantation, leverages the regenerative capacity and specific anatomy of the liver, resulting in a 5-year survival rate of approximately 70 to 80% [35,36]. However, up to 80% of patients experience liver tumor recurrence [37]. Ablation therapy is advised for patients with early-stage HCC (tumors ≤2 cm in size) or with 2- to 4-cm lesions that cannot be surgically removed due to anatomical constraints or the patient’s overall condition [3]. However, the scatter effect of local ablative therapy is associated with recurrence, with over 30% of patients experiencing recurrence or metastasis [38]. Transarterial chemoembolization (TACE), an IAT approach that takes advantage of the unique vascular structure of the liver, is preferred for intermediate-stage patients. However, viable tumor cells may remain after TACE, potentially leading to local recurrence and distant metastasis [39]. Patients with advanced disease initially receive systemic therapy [2]. Small-molecule chemotherapeutic agents target molecules involved in specific tumor growth-related signaling pathways, such as receptors for vascular endothelial growth factor (VEGF) and platelet-derived growth factor (PDGF). Immunotherapy, by harnessing immune cells within the TME, seeks to revive the immune system’s capacity to combat tumor cells by counteracting tumor-induced immunosuppression. Gene therapy and other biologics can also directly target tumor cells or their microenvironment, allowing more precise treatments. Despite significant academic advances in these treatments, only a few advanced therapeutic strategies have been successfully translated into clinical applications [40]. In conclusion, while various therapeutic options exist for different stages of HCC, their efficacy in completely eradicating the tumor may be limited. This challenge has inspired the exploration of novel therapeutic vehicles, with hydrogels emerging as a promising solution. As a drug delivery system, hydrogels offer multifaceted advantages, fully leveraging the unique anatomy of the liver and addressing the challenges of the TME. They are capable of encapsulating a broad spectrum of therapeutic agents, ranging from small molecules and bioactive substances to complex biomolecules such as proteins and nucleic acids, enabling localized delivery to the tumor site for sustained release [12,13]. Additionally, the stimulus responsiveness of hydrogels enables precise drug release in response to TME-specific stimuli [11], increasing the therapeutic effect while minimizing the impact on surrounding healthy tissues. Thus, the modulatory capacity and stimulus responsiveness of hydrogels render them a powerful tool for treating HCC.

## Hydrogel-Assisted Tumor Administration

Biomaterials can be classified based on their chemical composition, including polymers, metals, ceramics, or composites [41,42]. Hydrogels, which fall within the category of polymers, possess a distinctive 3D crosslinked polymer network structure comprising 2 main components: polymer chains and water [43]. They are capable of absorbing significant quantities of water into their interstitial spaces, continuously bonding water while preserving their network structure in an expanded, swollen state, resulting in a highly hydrophilic porous 3D network [44]. Accordingly, hydrogels demonstrate biological attributes similar to the high water content and perm eability found in tissues, supporting the influx of nutrients or the efflux of metabolites. Moreover, hydrogels exhibit notable adsorption capacity and biocompatibility and maintain substantial physical and chemical resilience under physiological conditions, mirroring the properties of human tissues. Notably, hydrogels are responsive to environmental stimuli and are biodegradable, and they expand and contract in response to aqueous conditions without considerable or irreversible harm to their structural integrity [45].

### Hydrogel classification

The creation and development of hydrogels involve a diverse range of polymers and crosslinking agents, categorized broadly into natural and synthetic types. Common natural polymers include collagen, gelatin, hyaluronic acid (HA), alginate (ALG), fibrin, and peptides [46]. They are inherently biocompatible, bioactive, and biodegradable but relatively low in stability and mechanical strength [47]. Synthetic hydrogels, consisting of synthetic polymers, provide greater flexibility in terms of tuning the mechanical properties. The most commonly used synthetic polymers include polycaprolactone (PCL), polyvinylpyrrolidone (PVP), polylactic acid (PLA), polyethylene glycol (PEG), and polyvinyl alcohol (PVA) [48]. Compared to natural hydrogels, synthetic hydrogels offer a longer service life, higher water absorption capacity, and greater gel strength. Additionally, synthetic polymers often feature well-defined structures that can be modified to achieve customized degradability and functionality [47]. The categorization and construction of hydrogels, including materials, technologies, and various physicochemical properties, are detailed in Table. Semisynthetic hydrogels combine the biological advantages of natural hydrogels with the mechanical properties of synthetic hydrogels because they consist of chemically modified natural polymers or combinations of natural and synthetic polymers as base materials, including PEG-conjugated fibrinogen or a combination of gelatin and albumin [49], methacryloyl-modified gelatin (GelMA) [50], and acrylate-modified hyaluronic acid (AcHyA) [51]. These hydrogels are characterized by adjustable physical and chemical attributes [52], such as strength, porosity, degradation pace, and drug release behavior, which are tailored to fulfill the specific requirements of liver cancer treatment.

**Table.** Diverse biomedical applications of hydrogels: Composition and physicochemical aspect

Hydrogel material	Crosslinking method	Bioactivity	Degradability	Network pore size	Viscoelasticity	Stiffness	References

Collagen	pH and temperature TG, Ca^2+^ PEG-diNHS Methacrylation/photopolymerization	Col-derived triple-helical ligands (e.g., GxOGER)-β1-containing integrins, RGD peptides	Collagenase, MMPs, plasmin, pepsin	Fibrous	Viscoelastic	10–250 Pa (G′)	[285,286]
Gelatin	TG, Ca^2+^ Methacrylation/photopolymerization Ferulic acid (FA) Conjugation/Laccase (02)	RGD-a5ß1 and avß3	Collagenase, MMPs, plasmin, pepsin	Macroporous	Viscoelastic	11–1,800 Pa (G′)	[287]
Hyaluronic acid (HA)	Acrylation/photopolymerization Host–guest interaction	Specifically reacts with CD44 receptor	Hyaluronidase, MMPs	–	Viscoelastic	1.3–10.6 kPa (E)	[288–290]
Alginate	Divalent cations (e.g., Ca^2+^ or Ba^2+^) Oxidation + gelatin	–	Chelator (e.g., sodium citrate, EDTA, etc.)	Nanoporous	Viscoelastic	0.5–3 kPa (G′)	[291,292]
Fibrin	Thrombin/Ca^2+^ PEG-diNHS	RGD-a5ß1 and avß3	Chymotrypsin, actinase, carboxylase, etc.	Fibrous	Viscoelastic	0–8 kPa (G′)	[293]
Agarose	Temperature	–	Hydrolysis	–	Viscoelastic	1–120 kPa (G*)	[294]
Polypeptide	Self-assembly	–	Enzyme	Fibrous	Viscoelastic	500–2,500 Pa	[295–297]
DNA	Base pairing	–	Nuclease	–	Viscoelastic	4–23 kPa (E′)	[298,299]
**Synthetic material**
PEG	Acrylation/photopolymerization Thiolation	–	Hydrolysis	–	Elastic	1–1,200 kPa (compression stress)	[300]
PVA	Calcium gluconate Glutaraldehyde, etc.	–	Hydrolysis	–	Elastic	3.7–30.2 kPa	[301–303]
PCL	Temperature	–	Hydrolysis	–	Elastic	5 MPa (compressive strength)	[304]
Polyacrylamide	Free radical copolymerization	–	Hydrolysis	–	Elastic	0.1–740 kPa	[305,306]
Polyurethane (PU)	Temperature	–	Hydrolysis	–	Elastic	0.68–2.4 kPa (E′)	[307,308]

Several injectable stimuli-responsive hydrogels targeting the complex TME of HCC are considered promising for drug delivery within tumors [14]. These hydrogels are highly sensitive to microenvironmental factors specific to HCC, such as unique enzyme activities, ROS levels, redox states, and pH fluctuations, and undergo volumetric phase changes, swelling/shrinking, or assembly/disassembly [11,53]. Such hydrogels can precisely control localized drug release and duration while maintaining easy injectability and good biodegradability [14]. To fabricate enzyme-responsive hydrogels, 2 primary strategies are used: utilizing inherently responsive natural polymers, including collagen, gelatin, HA, and fibronectin, and incorporating biomaterials that feature enzyme-sensitive linkages [54]. Drug release is triggered by specific enzymes, resulting in a highly precise on-demand drug delivery. Drug carriers can also be enzymatically activated to expose targeted ligands for subsequent internalization into specific cells. For tumor environments with high ROS levels, ROS-responsive hydrogels are designed to release therapeutic drugs in a precise and timely manner to attack tumor cells while minimizing the impact on healthy tissues [55]. Sulfide-containing polymers are the most frequently employed ROS-responsive materials in biomedical fields. Within an oxidizing microenvironment, these sulfide-bearing ether polymers transition from a hydrophobic (sulfide) state to a more hydrophilic (sulfoxide-sulfone) state. This phase transition can lead to carrier destabilization and thus drug release [56]. Redox-responsive drug delivery systems specifically target the increased oxidative stress in tumor cells, which use elevated glutathione (GSH) levels to respond to oxidative species in the intracellular environment [57]. By incorporating disulfide bonds, polymers with redox-sensitive side chains can be developed. The cleavage of these bonds disrupts the balance between hydrophobic and hydrophilic blocks, leading to drug release [58]. pH-sensitive hydrogels are obtained by conjugating charged pendant groups such as amino, sulfonyl, or carboxyl groups onto the polymer backbone [59]. These groups ionize at specific pH levels, leading to hydrogel swelling [60]. Such pH-responsive hydrogels exhibit relatively high stability at pH 7.4 and undergo partial hydrolysis at pH values encountered outside the cell membrane in tumors such as HCC, releasing the drug and thereby significantly inhibiting tumor growth [61]. With these combined strategies, we can not only enhance the performance of hydrogels as drug delivery vehicles but also introduce new therapeutic avenues for HCC treatment.

Hydrogels made from various polymers can provide tailored therapeutic platforms for the specific needs of liver cancer patients. Compared to traditional drug formulations, hydrogels present a number of advantages, including local targeting, sustained release, and stimuli responsiveness in drug delivery. Moreover, these materials offer excellent injectability, effective encapsulation of both hydrophobic and hydrophilic drugs as well as therapeutic cells, superior drug release profiles, and advantageous processing and molding properties [62,63]. Consequently, hydrogels hold great potential as a powerful tool in HCC treatment, offering patients more precise and effective therapeutic options.

### Multiscale hydrogels

Because of their unique physical properties, hydrogels are of particular interest for drug delivery. In liver cancer therapy, hydrogels can flexibly adapt to the shape of complex organs such as the liver through macroscopic design adjustments, including size and porous structure, along with their excellent deformability [64], significantly expanding their potential applications in drug delivery. The characteristic sizes of hydrogels range from the centimeter scale down to the subnanometer scale, covering a range from megagels (>100 μm) to microgels (0.5 to 10 μm) and nanogels (<200 nm). The macroscopic structure of hydrogels significantly influences their mode of entry into the human body [12]. The choice of delivery route for hydrogels in HCC therapy can be tailored to meet specific therapeutic requirements, with a focus on increasing therapeutic effectiveness and patient compliance. Additionally, the mechanism of drug release from the hydrogel is pivotal for achieving the intended therapeutic effect. The necessary duration of drug availability (short-term versus long-term) and drug release profile (continuous versus pulsed) are dictated by the particular therapeutic application. Moreover, it is imperative that hydrogels be engineered either to degrade after drug depletion, to preclude the need for surgical removal, or to be able to be recharged with the drug, to allow reuse [12]. Consequently, hydrogels, as a versatile and adaptable drug delivery system, not only improve the targeting and increase the efficiency of HCC treatment but also offer patients safer and more convenient treatment options.

#### Minimally invasive hydrogels for HCC

Local tumor delivery and IAT are frequently employed minimally invasive techniques for HCC. Local drug delivery systems concentrate the medication in the tumor area, minimizing the effects on healthy tissues by avoiding systemic circulation to prevent nontargeted interactions [65]. Hydrogels, recognized for their high drug loading capacity, stability, and responsiveness to environmental stimuli, play a crucial role in this approach. Owing to their considerable size, megagels present delivery challenges and are therefore employed in in situ delivery techniques. These methods include transdermal delivery, direct injection, or direct application to the surface of a surgical cavity, thereby amplifying the therapeutic impact [66]. For malignant tumors with complex anatomy, complete surgical removal is challenging. Even with postsurgical systemic chemotherapy, drugs often fail to successfully penetrate residual cancer sites, leading to poor efficacy [67]. High-performing megagels can be directly injected during surgery or sprayed onto the interior and surface of cancerous tissues after surgery, thus accurately targeting cancer cells, reducing the impact of drugs on normal tissues, and enhancing therapeutic efficacy [68]. Majumder et al. [69] introduced a surface-filled hydrogel (SFH) encapsulating microRNA nanoparticles with anticancer properties. This SFH stands out for its remarkable adaptability to shape, seamlessly filling gaps and fissures of varied configurations when sprayed or injected into complex anatomical locations. The most straightforward approach employs a slow-gelling system that allows the initiation of gelation ex vivo. Due to the gradual nature of this process, the solution can be administered before it solidifies. The gelation kinetics are ideally balanced to prevent the needle from clogging while also preventing dilution of the pregel solution by bodily fluids upon injection. Moreover, the development of temperature-sensitive systems that solidify at body temperature offers a promising avenue for in situ gelation. While most natural polymers, such as gelatin, gel upon cooling and therefore must be introduced into the body at temperatures above physiological levels, certain synthetic polymers, such as poly(*N*-isopropylacrylamide) (PNIPAm) and poly(ethylene oxide)-poly(propylene oxide)-poly(ethylene oxide) (PEO-PPO-PEO), exhibit reverse thermogelation: They solidify as temperatures increase [70,71] and remain flowable at room temperature, offering significant practical advantages [12]. For instance, Fan et al. [72] formulated a hydrogel comprising poloxamer 407, poloxamer 188, and the bioadhesive component carbomer 974 P. This hydrogel is characterized by its effective temperature sensitivity, which allows it to be directly applied to the surface of surgical cavities following cancer resection without adverse effects on surrounding healthy tissues. This approach has potential for preventing both tumor recurrence and the development of distant metastases. In particular, for HCC, leveraging the unique anatomical and physiological characteristics of the liver, especially its dual blood supply system, renders IAT therapy highly appropriate for liver cancer treatment. Even highly targeted drug delivery methods such as TACE can leave behind active tumor cells due to the complexity of the TME and the heterogeneity of HCC. However, exploiting the unique physical properties of hydrogels allows the creation of systems that can gradually release drugs at the tumor site, sustaining therapeutic effectiveness while reducing adverse effects on healthy liver tissues [73]. Furthermore, the appropriate size, antimigration characteristics, ease of administration, and degradability of hydrogels render them highly suitable for use as embolic agents [66]. Ablation therapy, a local treatment for liver cancer, destroys tumor cells through local heating. However, incomplete destruction of the tumor margin can lead to residual or recurrent tumors [74]. In contrast, hydrogels can precisely target the marginal area for treatment. This is due to a complex interaction of multiple mechanisms. First, the hydrogel material can be delivered directly to the tumor area through local injection, thereby avoiding systemic side effects and accurately covering complex and irregular tumor margin areas. Second, through chemical modification, hydrogels can bind specific tumor cell surface receptors or biomarkers in the microenvironment, thereby enhancing their targeting in the tumor margin region. Additionally, hydrogels can be engineered to respond to particular signals present within the TME, including low pH, elevated hydrogen peroxide concentration, or high enzyme activity. For example, Zheng et al. [75] developed a multifunctional hydrogel that enabled precise tumor margin ablation through local injection. Its excellent tissue retention ability and microenvironment-responsive design ensured efficient coverage and treatment of tumor margins. Moreover, hydrogels can adapt to changes in the TME caused by ablative therapy, such as reduced local blood flow and insufficient oxygen supply, to maintain the stability and efficacy of the drug. Additionally, as local therapy is not affected by tumor vascularity, drug delivery is not limited to better-perfused tumor areas. Compared to implants (wafers, rods, and films) and pellet-based drug delivery systems, injectable biodegradable hydrogels can facilitate minimally invasive, nonsurgical treatment and increase the retention of free or encapsulated drugs at the tumor site [76].

#### Oral hydrogels for HCC

Oral drug delivery systems have attracted considerable interest for the treatment of HCC owing to their advantages in enabling long-term dosing, increasing safety, offering convenience, reducing discomfort, and decreasing health care expenses [77,78]. Biocompatible hydrogels play a pivotal role in oral drug delivery because of their noninvasiveness and ability to transport a diverse array of substances, including drugs, cells, bacteria, and proteins, safely within the body [79]. Specifically, microgels have been instrumental in enhancing the loading and controlled release of hydrophobic drugs, which are crucial for targeting liver cancer cells. In HCC treatment, the transoral ingestion of drug-coated hydrogels facilitates rapid delivery of the active substance directly to the liver, resulting in increased local drug concentrations and reduced systemic side effects. The presence of microgels ensures sustained high mucosal permeability and stability within the gastrointestinal (GI) tract, prolonging the efficacy of the drug [66]. This therapeutic strategy effectively circumvents the first-pass effect, reducing the amount of drug metabolized during its initial pass through the liver [80]. Through the slow-release mechanism, the hydrogel can gradually release the drug at the optimal timing and intestinal location, increasing the amounts of active compounds that directly reach the liver and take effect. This approach improves the bioavailability of the drug, reduces potential side effects due to excessive metabolism, and offers a safer and more effective therapeutic option for HCC patients. Furthermore, stimulus-responsive hydrogels, which alter their network structure, swelling behavior, permeability, or mechanical properties in response to environmental stimuli, play a crucial role in enabling precise drug release, a key factor in liver cancer therapy and oral drug delivery [81]. pH-responsive hydrogels, in particular, are vital for oral drug systems. They undergo minimal swelling in the acidic stomach environment, keeping the drug encased and intact, and then swell extensively upon reaching the neutral pH of the intestine, promoting rapid drug diffusion [12]. A limitation of the swelling control system is the relatively slow response of macroscopic hydrogels, which is attributable to slow water diffusion. For a hydrogel measuring 1 mm, swelling and drug release modifications might require tens of minutes. A quicker response can be achieved by reducing the hydrogel’s size or by creating interconnected macropores within the hydrogel structure, thereby shortening the diffusion length.

#### Intravenous hydrogels for HCC

Intravenous drug administration, a common route, delivers drugs directly into the circulatory system, where they rapidly reach all parts of the body. Because hydrogels are injectable, biocompatible, and capable of modified drug release, they are particularly advantageous for intravenous delivery in HCC therapy [12]. Nanogels, which combine the benefits of nanotechnology and hydrogels, stand out in this context. They are structurally akin to natural tissues, offering stability, an extended lifecycle, and minimal biotoxicity. Additionally, their sub-200-nm size provides a very large surface area, high efficiency in drug loading, and the ability to target cancer cells through specific ligand binding [66,82]. The size of nanogels allows them to efficiently permeate vessel walls, particularly within the liver’s sinusoidal capillaries, which are characterized by fenestrated endothelial cells lacking a basement membrane. This enhanced permeability, along with the liver’s dual blood supply from the hepatic artery and portal vein, contributes to the enhanced permeability and retention (EPR) effect, facilitating the accumulation and retention of therapeutic agents within the liver [83,84]. Furthermore, hydrogels facilitate the development of activation-modulated and site-targeted drug delivery systems, thereby presenting promising avenues for HCC treatment [85]. Activation-modulated systems release drugs in response to specific triggers, such as environmental stimuli, including light, temperature, or magnetic fields, or internal factors, such as enzyme activity, redox conditions, and pH variations. This precision ensures drug deployment at the most opportune time and location. Targeted delivery systems utilize mechanisms such as ligand–receptor interactions or antibody-mediated targeting to increase drug localization at the cancer site, improving treatment outcomes and reducing systemic side effects. A notable example is the work by Yang et al. [86], who developed a dual-drug delivery system combining a nanogel with an injectable water gel (NHG). This system was designed for the sequential release of cobutatin-A4 phosphate (CA4P) and doxorubicin (DOX) to achieve synergistic antiangiogenic and anticancer effects. The injectable hydrogel facilitated the immediate release of CA4P, while the pH- and redox-responsive nanohydrogel promoted the sustained release of DOX. Laboratory tests showed that DOX and CA4P could be released over periods of up to 14 d and 80 h, respectively, indicating the system’s capacity for controlled, sequential drug release. Animal studies have underscored the marked effectiveness of this system with only a one-time administration. This innovative approach not only improves drug targeting and efficacy but also significantly promotes the development of more tailored and efficient strategies for liver cancer treatment, leveraging the integration of therapies such as small-molecule drugs, immunotherapy, or other therapies to design new paths in personalized medicine.

## Small-Molecule Drug Hydrogels for HCC

Cytotoxic drugs function by remodeling the ECM [87], inhibiting neovascularization [88], and stimulating inflammatory responses [89], thereby altering the TME. In contrast, targeted drugs act on tumor cells by specifically inhibiting growth factor receptors or signaling proteins, blocking angiogenesis, or affecting metabolic pathways [90] to alter the TME and consequently inhibit tumor growth. Prior to the advent of sorafenib (SOR), conventional cytotoxic chemotherapy was the mainstay treatment for patients diagnosed with advanced HCC. However, intensive research efforts aimed at elucidating the mechanisms underlying drug resistance in HCC have demonstrably improved the median survival associated with this therapeutic approach, extending it to approximately 7.4 months [91]. Despite the extended median survival, the efficacy of this treatment is still hampered by limited efficacy and significant side effects [92]. In contrast, SOR, the first clinically validated targeted agent for advanced HCC, has been the standard of care for more than a decade [93]. The subsequent approval of lenvatinib further cemented the role of small-molecule targeted agents in the first-line treatment of advanced HCC [94]. However, targeted drug therapy can induce controllable adverse events [95], such as hypertension and diarrhea, and treatment interruption due to adverse effects occurs in approximately 9% of patients receiving lenvatinib patients and 7% of patients receiving SOR [96]. As an emerging drug delivery system, hydrogels offer multiple advantages, including enhanced drug bioavailability, reduced adverse events, and sustained drug release, potentially reducing the risk of treatment interruption [97,98]. This section explores the progress of using hydrogel-loaded small-molecule chemotherapeutic agents in HCC treatment and examines how this delivery system can be optimized through scientific design strategies.

### Nontargeted small-molecule hydrogels for HCC

**Fig. 4.**
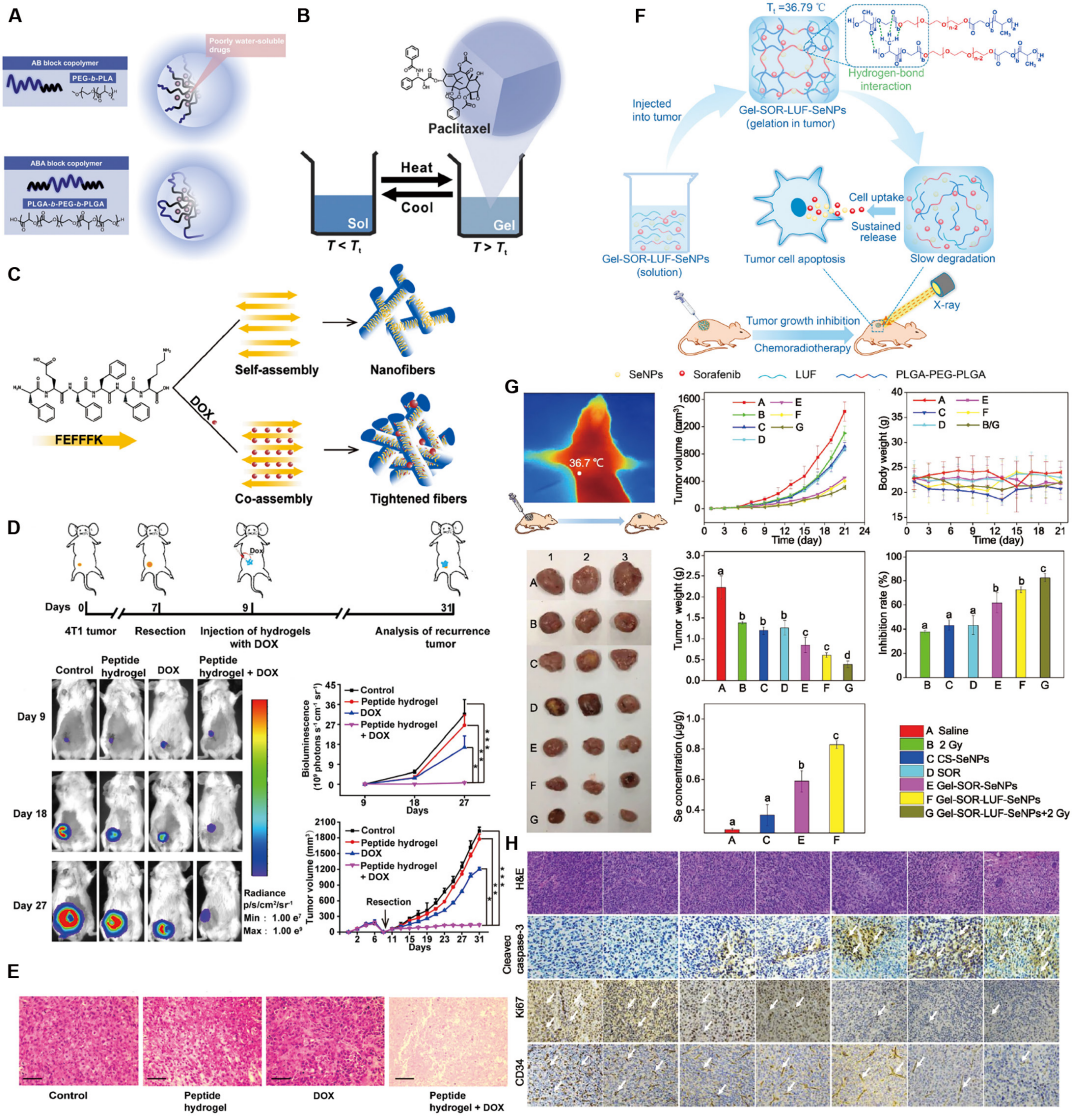
Hydrogel-assisted tumor small molecule. (A) PEG-b-PLA and PLGA-b-PEG-b-PLGA block copolymers for drug delivery. (B) Oncogel. Reproduced with permission [100]. Copyright 2015 Elsevier BV. (C) Molecular structure of the peptide FEFFFK self-assembled into a reverse parallel β-sheet arrangement. DOX affects the aggregation of nanofibers within the hydrogel matrix. (D) Hydrogel loaded with DOX reduced mammary tumor recurrence in mice. (E) Hematoxylin and eosin (H&E) staining of tumor sections after treatment. Reproduced with permission [101]. Copyright 2018 American Chemical Society. (F) Schematic representation of the proposed Gel-SOR-LUF-SeNPs as a novel synergistic anticancer thermosensitizer. (G) In vivo antitumor effects of Gel-SOR-LUF-SeNPs combined with x-rays. Saline, x-rays (2 Gy), CS-SeNPs (5.0 mg/kg Se), SOR (10 mg/kg), Gel-SOR-SeNPs (5.0 mg/kg Se and 10 mg/kg SOR), and Gel-SOR-LUF-SeNPs (5. 0 mg/kg Se and 10 mg/kg SOR) were tested, and the combination of Gel-SOR-LUF-SeNPs (5. 0 mg/kg Se and 10 mg/kg SOR) and x-rays (2 Gy) after 21 d showed the greatest effect. (H) Ki67, CD34, and cleaved caspase-3 expression and H&E staining of tumor tissues after treatment with different drugs for 21 d. Ki67-positive, CD34-positive, and cleaved caspase-3-positive proliferating cells were stained brown. Reproduced with permission [110]. Copyright 2019 Elsevier Ltd.

The principal aim of liver cancer treatment is the precise delivery and release of drugs at specific target sites within the body, with a concurrent reduction in nontargeted drug deposition, to increase therapeutic effectiveness [99]. Hydrogels can undergo an in vivo transition from sol to gel, thereby increasing the accuracy of dosing, improving the distribution of therapeutic agents, and enhancing drug penetration within tumor tissues [48]. Therefore, the selection of appropriate hydrogel materials is pivotal for increasing the efficacy of cytotoxic drug delivery systems. For example, PLGA-b-PEG-b-PLGA hydrogel, which is marketed under the name Regel, has undergone extensive investigation for both systemic and localized drug delivery applications (Fig. 4A). It has been successfully integrated with various drugs, including paclitaxel (PTX) and cyclosporine A, significantly improving their aqueous solubility at low temperatures and increasing their chemical stability in gel form at body temperatures. For example, Oncogel, containing 6.0 mg/ml PTX in Regel, with a polymer weight/water weight of 23% (Fig. 4B), is an optimal hydrogel material for delivering cytotoxic drugs. This optimization enables Regel to sustain drug release for up to 1 to 2 weeks, offering a highly efficient method for delivering nontargeted chemotherapeutic drugs [100]. Additionally, a hexapeptide (FEFFFK)-based hydrogel system with encapsulated DOX (Fig. 4C) demonstrated superior drug release compared to that of free DOX in animal models. At 1 and 4 d after injection, the DOX levels in the tumors in the hydrogel group were 1.6 and 1.8 times greater, respectively, than those of free DOX, while the DOX levels in the plasma were 2.3 times greater. After the administration of DOX-loaded hydrogels, the DOX levels in both tumors and plasma gradually decreased, but DOX remained detectable in tumors at 20 d after treatment, indicating substantial antitumor potential (Fig. 4D and E) [101]. Furthermore, hydrogels offer significant additional advantages due to their characteristic properties. A notable example includes a hydrogel composed of *N*-carboxyethyl chitosan (CEC) and dibenzaldehyde-capped polyethylene glycol (PEGDA) with encapsulated DOX, which demonstrated remarkable pH responsiveness, self-healing capabilities, and shear-thinning properties [102]. These characteristics promote effective drug release within the acidic TME, prolong the lifespan of the hydrogel, support the use of less invasive application techniques, and increase drug encapsulation efficiency. The outcomes indicate that this injectable, self-healing, pH-responsive hydrogel is an optimal vehicle for delivering anticancer drugs specifically tailored for HCC therapy.

Although hydrogels have shown remarkable potential and versatility as delivery materials for cytotoxic drugs in laboratory studies, they present numerous challenges in clinical applications. To date, research findings on hydrogel delivery systems have not been extensively translated into clinical practice [68]. For instance, hydrogel delivery systems for anticancer drugs such as PTX, cisplatin, and methotrexate in HCC treatment remain in the preliminary research stage, and their specific advantages and limitations require further elucidation [103]. To advance clinical and translational applications, future studies must prioritize safety and develop cost-effective and efficient synthesis methods for high-quality hydrogel materials. Additionally, challenges such as unstable drug release kinetics, complex hydrogel material fabrication, and multidrug resistance in HCC [104,105] must be addressed.

### Targeted small-molecule hydrogels for HCC

With the widespread adoption of targeted agents such as SOR and lenvatinib, liver cancer treatment strategies have undergone a significant transformation [93,94]. These drugs employ an active targeting mechanism, with carefully designed drug particles (with design characteristics including material type, particle size, charge, functional groups, and targeting molecules) circulating in the bloodstream for a prolonged period and specifically recognizing and targeting cancer cells, thus triggering effective drug release [106]. Hydrogel materials used as drug carriers in HCC therapy have several abilities that complement small-molecule targeted drugs, including providing more efficient drug delivery to the tumor site [107], providing local adjuvant therapy to reduce systemic side effects [108], and increasing drug bioavailability through tunable physical properties [109]. These properties further optimize drug distribution in the body and enhance therapeutic efficacy.

The Gel-SOR-LUF-SeNP thermosensitive nanosystem developed in a recent study successfully integrates SOR and selenium nanoparticles (SeNPs) into PLGA-PEG-PLGA hydrogels, providing a comprehensive solution for HCC treatment (Fig. 4F). The Gel-SOR-LUF-SeNPs exhibited good degradation performance and significantly reduced the initial burst release (46%) within 3 d, resulting in the sustained release of SOR for more than 15 d (Fig. 4G and H). This innovative approach successfully ensures localized, controlled, and sustained drug release, combining local and systemic therapeutic strategies to optimize treatment efficacy [110]. Amlotinib hydrochloride (AL) was successfully encapsulated in a HA hydrogel to create a complex named AL-HA-Tyr, which significantly increased the antitumor effect of AL and effectively mitigated its side effects, such as hypertension and diarrhea, thereby offering a safe and effective cancer treatment strategy [111]. However, the integration of hydrogels with small-molecule targeted drugs for HCC treatment still presents numerous challenges. Existing studies indicate that the in vivo matrix microenvironment is finely and dynamically regulated in terms of biochemistry and biomechanics, potentially affecting cell behavior and therapeutic efficacy. Although several dynamic hydrogel systems have been developed, their application has been focused primarily on drug delivery rather than on comprehensively addressing this complexity. Consequently, we have not yet achieved a comprehensive understanding of cell behavior and associated signaling cascades within hydrogel systems, constraining our ability to predict therapeutic efficacy and contributing to the complexity of precision medicine [48]. Moreover, despite the considerable potential of hydrogels for multifunctionality, the mechanisms underlying their interactions with small-molecule targeted drugs have yet to be thoroughly investigated, limiting their widespread application in clinical trials. Additionally, regulatory agencies are cautious about the safety and efficacy of these novel combination therapies, subjecting them to a rigorous review and approval process that has slowed their market introduction [112]. In conclusion, combining advanced hydrogel systems with targeted chemotherapeutic agents is expected to foster more precise and personalized cancer treatment options, offering patients more tailored treatment choices [113].

## Immunotherapy Hydrogels for HCC

The application of immunotherapy in the treatment of HCC represents a significant advancement in the field of oncology. The distinctive TIME of HCC has prompted the utilization of immuno-oncology drugs [114]. The TIME not only provides essential biochemical and physical support for tumor growth but also plays a crucial role in immune evasion and treatment resistance in tumors [115]. The TIME in HCC, which comprises various immune cells, ECM components, cytokines, and chemokines, fosters an immunosuppressive and proinflammatory milieu that supports the progression and metastasis of HCC and poses significant challenges to therapeutic interventions [25]. It is noteworthy that immunotherapy has shown considerable promise in improving outcomes for HCC patients, as exemplified by strategies that yield increased survival rates and more positive prognoses [116]. A landmark combination therapy involving atezolizumab [an anti-PD-L1 monoclonal antibody (mAb)] and bevacizumab (an anti-VEGF mAb) demonstrated a 29.8% overall response rate (ORR) and extended survival by 5.8 months in patients with unresectable HCC. This combination was found to be more effective than SOR, as highlighted by the results of the IMbrave150 trial [117,118]. However, tumors can achieve immune evasion through various mechanisms, such as creating an immunosuppressive microenvironment or manipulating cellular immune responses [119]. Furthermore, the pharmacokinetics and specificity of immunotherapeutic agents can often result in significant immune-related adverse effects and restricted effectiveness [120]. Hydrogels have emerged as a promising solution to these challenges, offering controlled and targeted drug delivery systems that can increase the efficacy and applicability of immunotherapy [97,121]. By facilitating controlled release and precision targeting, these technologies can significantly increase the effectiveness of immunocombination therapies, reduce the frequency of drug administration, lower treatment costs, and improve patient compliance [122]. This section explores the progress of hydrogel-loaded immunotherapeutic agents, including immune checkpoint blockers, adaptive cell therapies, and immunovaccines, in liver cancer treatment.

### Immune checkpoint blockade hydrogels for HCC

**Fig. 5.**
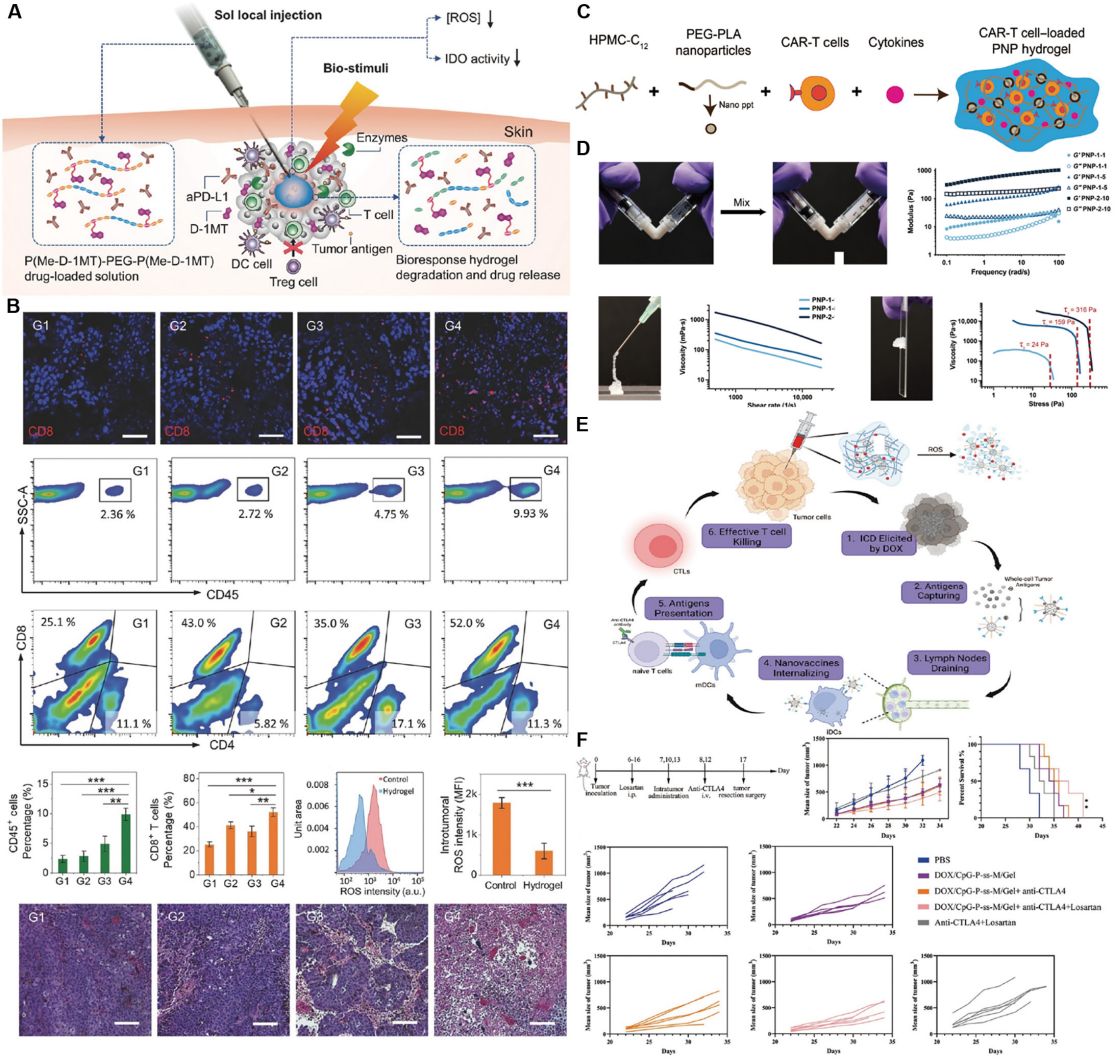
Hydrogel-assisted tumor immunotherapy. (A) Schematic illustration of localized hydrogel formation, biostimulation-triggered drug release, and synergistic immunotherapy. (B) Assessment of the in vivo antitumor efficiency of P(Me-D-1MT)-PEG-P(Me-D-1MT) hydrogels. Reproduced with permission [127]. Copyright 2018 Wiley-VCH. (C) Formation of PNP hydrogels to encapsulate CAR-T cells and stimulatory cytokines by the self-assembly of dodecyl-modified hydroxypropylmethylcellulose (HPMC) and degradable block polymer nanoparticles. (D) The rheological properties of PNP hydrogels make them injectable and capable of forming reservoirs. Reproduced with permission [138]. Copyright 2022 American Association for the Advancement of Science. (E) Mechanism of the in situ nanovaccine system: DOX induces immunogenic cell death (ICD) and generates whole-cell tumor antigens; tumor antigens are captured by CpG-P-ss-M and drained to lymph nodes; the nanovaccine is internalized by DCs and promotes their maturation, which ultimately increases T cell activation, proliferation, and intratumoral infiltration. (F) Combinatorial therapy suppresses postoperative tumor relapse. Reproduced with permission [152]. Copyright 2023 Wiley-VCH.

The pivotal role of immune checkpoints in immunosuppression, which is crucial for maintaining self-tolerance and preventing autoimmune diseases, has been highlighted by functional research [123]. The PD-1/PD-L1 and CTLA-4/B7 pathways, as key negatively regulated immune checkpoints, have been extensively targeted in clinical research in recent years, aiming to disrupt these pathways to promote immune responses against tumors [124]. Although immune checkpoint blockade (ICB) has demonstrated superiority to other therapeutic options for many advanced tumors, only a small fraction of patients have experienced satisfactory outcomes. Additionally, ICB therapies are associated with a spectrum of immune-related adverse events (IrAEs), ranging from mild to potentially life-threatening conditions [125]. Hydrogel-based drug delivery systems present a novel approach for optimizing the delivery and efficacy of ICB drugs. By adjusting the pharmacokinetics and pharmacodynamics of drugs, these systems can enhance therapeutic outcomes and reduce systemic side effects. Furthermore, they can be combined with other immunomodulatory agents, potentially transforming the TME from immunologically “cold” (low immune cell infiltration and response) to “hot” (high immune cell infiltration and active immune response), thereby achieving synergistic therapeutic effects [124]. A noteworthy application by Yu et al. [126] involved the development of a peptide-based injectable gel depot for the sustained release of anti-PD-L1 (aPD-L1) and dextro-1-methyltryptophan (D-1MT), along with the modulation of ROS levels within the TME, aiming to improve therapeutic effectiveness. This approach highlights the dual function of hydrogels: as a delivery medium for the efficient transport of therapeutic agents and as a modulator of the TME to increase treatment efficacy. In vivo studies, such as those demonstrating the antitumor effectiveness of intratumoral injection of Gel-SOR-LUF-SeNPs in HepG2 xenograft nude mice compared to that of free drugs, as shown via x-ray imaging (Fig. 5A), emphasize this dual capability. In another innovative study, researchers created Zeb-aPD1-NPs-Gel, an in situ-formed hydrogel that responds to the high ROS levels and acidic conditions of the TME, releasing approximately 75% of aPD1 (Fig. 5B) [127]. This gel promotes exposure to tumor-associated antigens (TAAs), diminishes suppressive immune cells, counteracts the immunosuppressive tumor microenvironment (ITM), and increases PD-L1 expression. Coupled with the role of aPD1 in blocking PD-L1/PD-1 interactions, this dual-responsive system triggers a strong antitumor immune response. Additionally, assessments of body weight changes and histological analyses of major organs revealed no significant toxicity of Zeb-aPD1-NPs-Gel in comparison to controls, indicating a satisfactory safety profile. These studies illustrate the potential of hydrogel delivery systems to revolutionize the application of immunotherapy in HCC treatment, providing a foundation for more effective, personalized cancer therapy strategies.

Although advances in the locally targeted delivery of ICB agents have led to significant advances in cancer therapy, several hurdles remain to be overcome to facilitate their clinical application. One of the primary challenges involves synthetic delivery systems, where there is a critical need to optimize the loading efficiency, responsive drug release, and degradation kinetics to maximize therapeutic benefits. Despite the relatively high availability of ICB formulations designed for these systems, their practical use may be limited by the anatomical location of tumors, especially those situated in deep tissues, necessitating further advances in delivery methodologies [124]. Moreover, as the application of ICB in oncology expands, the number of patients receiving an increasing array of ICB drugs is increasing. The association of ICB therapy with specific biomarkers has the potential to improve patient selection, leading to more consistent and durable responses. However, the identification and validation of such biomarkers for HCC remain incomplete, representing a significant gap in the current treatment landscape [128]. Thorough investigation of IrAEs through comparative analysis between systemic and locally targeted therapies is imperative to underscore the potential benefits of localized treatments in diminishing side effects [124,129].

### Adaptive cell therapy hydrogels for HCC

Adaptive cell therapy (ACT) employing effector cells is a form of passive therapy in which lymphocytes are sensitized and/or expanded in vitro before being reinfused into the patient. In ACT, large quantities of antitumor lymphocytes (up to 10^11^) can be readily cultured and screened in vitro for the robust recognition of tumors and for mediating the effector functions necessary for cancer regression. In vitro activation liberates these cells from inhibitory factors present in vivo. Moreover, ACT can modify the host microenvironment prior to cell transfer to promote antitumor immunity [130]. The cells employed in ACT include a variety of immune cells, such as lymphokine-activated killer (LAK) cells, cytokine-induced killer (CIK) cells, NK cells, tumor-infiltrating lymphocytes (TILs), and redirected peripheral blood T cells, which are predominantly engineered to express either tumor antigen-specific T cell receptors (TCRs) or chimeric antigen receptors (CARs) [130,131]. Among these, emerging preclinical and clinical evidence has revealed CAR-T cell therapy as a promising approach for treating HCC patients [132,133]. Moving forward, the focus will be on the application of hydrogel materials in combination with CAR-T cell therapy.

CAR-T cell therapy, distinguished by its ability to specifically target TAAs without being restricted by the major histocompatibility complex, is poised for refinement [132]. Ideal CARs should effectively and specifically distinguish tumors from normal tissues based on the expression of target antigens and rapidly migrate to tumor sites [134]. Nonetheless, unlike hematologic malignancies, solid tumors present obstacles such as variable antigen expression variability, the difficulty of CAR-T cell infiltration into tumors, and tumor resistance to CAR-T cell therapy [135]. Hydrogel-based drug delivery systems have emerged as a solution for enhancing the precision and controlled release of CAR-T cell therapies, focusing their effects on the intended cells while reducing unintended impacts [136]. An example of this approach involves the use of a hydrogel formed from acrylate-modified HA as a medium for CAR-T cell administration [137]. This system demonstrated the capacity for the sustained release of CAR-T cells in vivo, releasing 30% of the cells within the initial week and achieving a total of 50% release by the end of the following period. To precisely monitor the distribution, functionality, and success of CAR-T cells in vivo, the cells were tagged with luciferase. This tagging facilitated the effective tracking of the remote impacts and enduring presence of CAR-T cells. These findings indicate the potential of this method for treating metastatic tumors and lay the groundwork for refining CAR-T cell therapy approaches in future research.

Currently, CAR-T cell therapy is predominantly applied intravenously and has proven effective for treating hematological malignancies because it allows T cells to easily locate and eliminate cancer cells [138]. Regrettably, contemporary therapeutic approaches often require substantial amounts of CAR-T cells for efficacy, leading to complicated and labor-intensive in vitro expansion processes. This requirement potentially compromises the effector potential of the cells in treating metastatic conditions [139,140]. Additionally, the conventional systemic administration of CAR-T cells has reduced efficacy against solid tumors due to difficulties in the targeting, infiltration, and proliferation of CAR-T cells within the immunosuppressive environment of solid tumors [141]. In contrast, innovative delivery techniques, particularly those utilizing biomaterial scaffolds augmented with stimulatory molecules, have shown promise in increasing the impact of systemically administered therapies on solid tumors. These methods promote T cell proliferation at the site of administration, thus increasing tumor penetration and overall therapeutic effectiveness [142,143]. However, the production of biomaterial scaffolds suitable for ACT often involves intricate manufacturing processes that are not easily adaptable to all solid tumor types [144]. Moreover, these scaffolds typically require invasive surgical placement to reach the tumor site, which limits their applicability, especially in postsurgical scenarios [137,142,143,145]. There is evidence that local delivery using physically crosslinked hydrogel scaffolds can increase cell survival during injection and cell retention in the targeted area, promoting localized expansion of the introduced cells [143,144]. In response, Grosskopf et al. [138] developed a self-assembling, injectable biomaterial platform for CAR-T cell delivery utilizing polymer-nanoparticle (PNP) hydrogels (Fig. 5C and D). These hydrogels are designed to facilitate simple, direct injection and create a temporary inflammatory niche in vivo, which significantly promotes CAR-T cell proliferation and activation and markedly increases the efficacy of solid tumor treatments in mouse models [138]. Additionally, these hydrogels are composed of highly scalable chemicals [146] and can be prepared under mild conditions, allowing CAR-T cells and cytokines to be encapsulated without undergoing alteration.

The use of cellular therapies for solid tumors, especially CAR-T cells, offers promising prospects for treating HCC and is deemed suitable for not only because of the lack of other effective therapies but also because it is suitable for tumors with complex biological characteristics [134]. In situ CAR-T cell delivery is an effective method for increasing antitumor effects, and the use of hydrogel materials as a delivery modality offers several advantages, including (a) easy implantation without the need for surgical procedures, (b) tunable physical and chemical properties, and (c) the potential to expand the delivery device to have multiple functions. In conclusion, further investigation is warranted into the potential of hydrogel materials combined with advanced immune cell therapy.

### Tumor vaccine hydrogels for HCC

Cancer vaccines are categorized into 4 principal categories: tumor or immune cell-based vaccines, peptide-based vaccines, viral vector-based vaccines, and nucleic acid-based vaccines [147]. These vaccines, as an alternative form of immunotherapy, not only possess preventive potential but also exhibit specificity, safety, and tolerability in treatment [148,149]. However, the efficacy of tumor vaccines can be hindered by the differential expression of target antigens between tumor cells and healthy cells. HCC patients exhibit a poor response to tumor vaccines due to the significant heterogeneity of tumor cells [150]. Furthermore, clinical trials have indicated that tumor vaccines are more effective in patients with early-stage tumors and less effective than anticipated in patients with advanced tumors [151]. The performance of tumor vaccines is influenced by several critical factors, such as antigen selection, the precision of vaccine delivery, and the immunosuppressive nature of the body’s microenvironment [152]. Advances in understanding the range of TAAs, the body’s innate immune responses, and new antigen delivery methods have significantly contributed to improving vaccine designs [153].

The hydrogel design enables the gradual in vivo degradation of the tumor vaccine, facilitating the systematic release of immunoreactive molecules. This slow-release process is instrumental in recruiting and activating immune cells, notably DCs, and the degradation and release rates are adjustable through modifications to the polymer network and concentration of its components [154]. Zhang et al.’s [152] research has led to an innovative in situ tumor nanovaccine system incorporating 3 key elements: DOX, the nanovaccine carrier CpG-P-ss-M, and a ROS-responsive PVA-TSPBA (3-(triethoxysilyl)propylboronic acid) hydrogel. This hydrogel is designed to respond to ROS levels, enabling the controlled release of its contents. Upon encountering ROS, the hydrogel releases DOX and CpG-P-ss-M in a concentration-dependent manner, which facilitates the in situ preservation of whole-cell tumor antigens. The nanoparticles are then released to bind tumor antigens and codeliver these antigens along with adjuvants to DCs, resulting in a robust antitumor immune response (Fig. 5E) and curbing postoperative tumor recurrence (Fig. 5F). The use of hydrogels as a vaccine delivery platform not only addresses the challenge of vaccine delivery but also optimizes the immune response by the controlled release of immunoreactive molecules. This in situ vaccine strategy is straightforward and easy to apply, bypassing the need for complex steps such as tumor antigen identification and in vitro antigen loading, as well as decreasing the substantial costs involved. Moreover, the topical application of this system results in minimal immunotoxicity to systemic tissues. This approach also significantly promotes tumor vaccine delivery to lymph nodes, increases antigen uptake, and stimulates immune activation, presenting a potent and cost-effective strategy for cancer immunotherapy.

Despite significant strides in cancer vaccine development, translating these vaccines into efficacious treatments through clinical trials has encountered hurdles, notably due to the vast diversity of tumor antigens and the typically low ensuing immune response [148]. Hydrogels have demonstrated considerable potential as vaccine delivery vehicles, particularly for immunomodulation and targeted delivery. However, additional research is necessary to successfully translate these technologies into clinical applications, particularly in overcoming immunosuppression in the TME [154,155]. In summary, the use of hydrogels as delivery platforms for cancer vaccines represents a novel and effective approach for optimizing immune responses and pioneering new avenues for cancer immunotherapy.

## Gene Therapy Hydrogels for HCC

The use of gene therapy, which involves the introduction of genetic material to patients with the aim of modifying cell function, has become increasingly prevalent in cancer treatment [156]. The liver has long been a preferred target for in vivo gene therapy [157] because the liver is critical for the synthesis of plasma proteins and proteins involved in numerous metabolic pathways. Furthermore, liver-expressed transgenes have been demonstrated to foster tolerance to transgene products [158,159]. Moreover, the effectiveness of hepatic gene therapy has been validated through numerous animal models and clinical trials [160–162]. The success of gene therapy for HCC depends on 3 critical factors: an effective gene delivery system, the identification of suitable gene targets, and the selection of appropriate delivery methods [163]. The efficient transportation of genes to the intended tissues or cells, a task facilitated by gene delivery vehicles, commonly referred to as vectors, is central to this approach. These vectors are broadly classified into 2 main groups: viral vectors and nonviral vectors [164]. New genetic information is conveyed into target cells, driving efficient expression of therapeutic molecules without disturbing the underlying regulatory mechanisms. Genetically modified cells must be numerous enough to reverse the disease, evade immune detection, and survive long-term or must be capable of transmitting altered information to progeny to sustain therapeutic efficacy [165]. Both intravenous and oral administration are convenient, noninvasive, and widely utilized in gene therapy. However, systemic administration poses a significant risk of off-target effects and necessitates larger doses than local administration for therapeutic efficacy, limiting its use in tissue-specific diseases [166]. Hydrogel systems offer several advantages over systemic delivery, including high permeability, low toxicity, and the capacity to promote gene regulation for enhanced biosafety [167]. Additionally, hydrogel systems sustain a high localized concentration of the vector, thereby overcoming the mass transport limitations of gene delivery. This approach significantly increases the colocalization of target cells and vectors and consequently the efficiency of gene transfer [168]. The high water content of the medium and the mild conditions under which the gel forms help to maintain the activity of the vector and protect it from potential damage. These features make it an optimal choice for gene delivery applications [169].

### Viral vector hydrogels for HCC

**Fig. 6.**
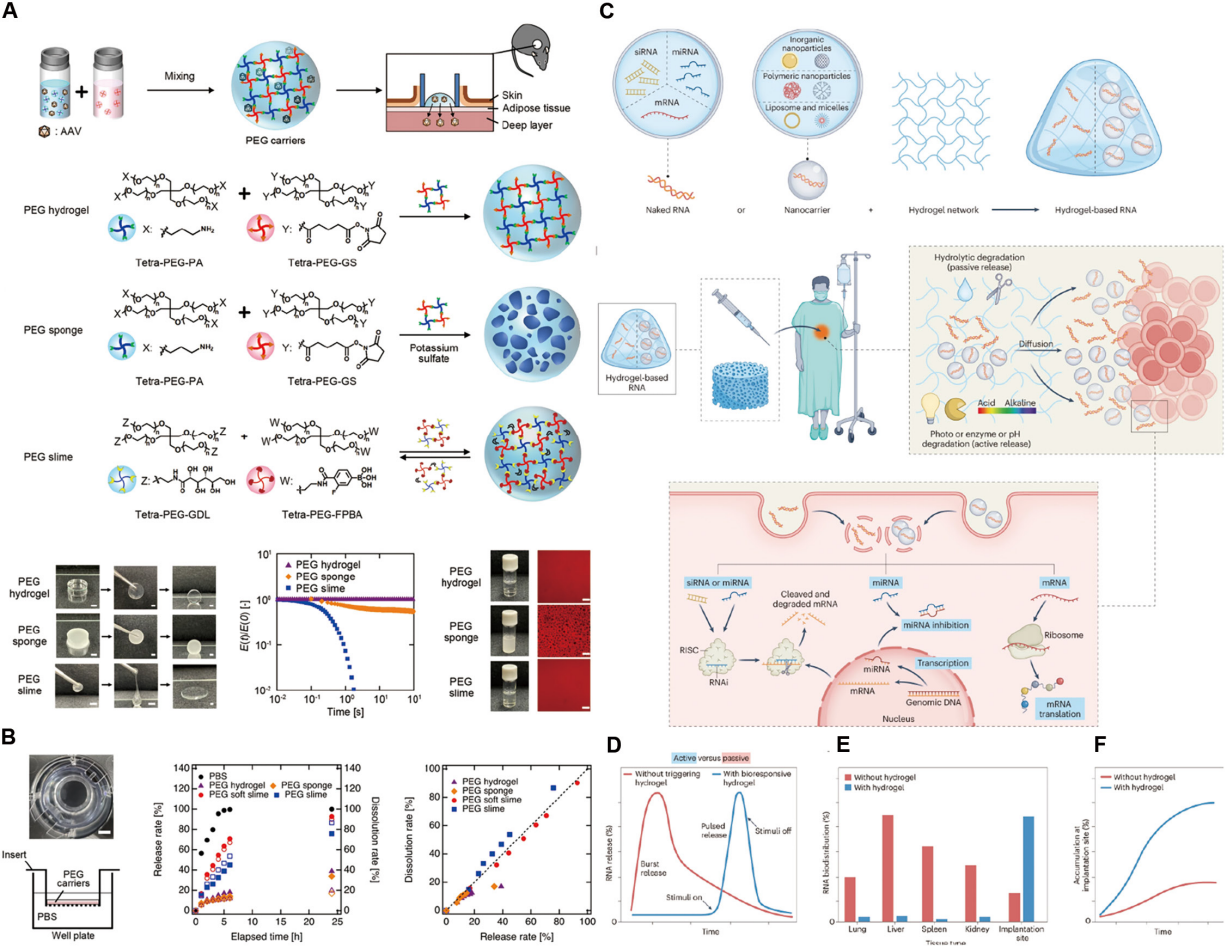
Hydrogel-assisted tumor gene therapy. (A) Conceptual diagram of an AAV encapsulated in a PEG vector and its characteristics. (B) In vitro evaluation of PEG vectors: silica nanoparticles as a model for virus lysis and diffusion. Reproduced with permission [176]. Copyright 2023 Springer Nature. (C) Functional hydrogels for the loading and delivery of RNA. (D) Schematic release profiles of encapsulated naked RNA and/or RNA nanocarriers. (E) Schematic representation of the biodistribution profile of RNA therapeutics administered in the naked form or in combination with a hydrogel system. (F) Schematic of the localized accumulation profile at the implantation site of the payload in the naked form or in combination with a hydrogel system. Reproduced with permission [167]. Copyright 2023 Springer Nature.

Modern viral vector-based gene therapy employs in vivo techniques to deliver therapeutic genes to patients using vectors derived from retroviruses, adenoviruses (Ads), or adeno-associated viruses (AAVs) [164]. The utilization of viruses as gene delivery vectors capitalizes on their natural capacity to infect cells and effectively transport genetic material into host cells [170]. The first gene therapy trial utilizing AAV for HCC, as described by Su et al. [171], employed the HSV-tk gene under the control of an α-fetoprotein (AFP) enhancer and an albumin promoter. This research demonstrated that viral vector-mediated gene delivery could be achieved through various routes, including the hepatic artery [172,173], portal vein, bile ducts, or direct liver injections. Clinical evidence of the efficacy of intratumoral injections of adenoviral vectors in HCC treatment has been supported by positron emission tomography imaging [174]. However, the potential of viral vectors to induce oncogenicity and immunogenicity remains a significant challenge for their use in gene therapy [175]. In response, hydrogel-based gene delivery systems have surfaced as innovative avenues in gene therapy research for HCC, as shown in Fig. 6A and B. A mixture of PEG with an equal volume of tetrapolyethylene glycol with a crosslinking function can be used as a carrier to encapsulate AAV for in situ application [176]. PEG hydrogels regulate the expression of transgenic vectors by providing a controlled release system that encapsulates and gradually releases viral vectors at the target site. The crosslinking density and degradation rate of the PEG hydrogel can be adjusted to tailor the release kinetics, ensuring sustained and localized transgene expression. This method prevents the rapid diffusion of vectors, reduces off-target effects, and minimizes immune responses. Moreover, the hydrogel matrix protects the encapsulated vectors from degradation, improving the overall stability and efficiency of gene delivery, as observed in studies involving AAV encapsulation within PEG hydrogels, as a way to overcome barriers to gene therapy. In contrast to conventional viral vectors, PEG hydrogels and their variants (e.g., sponges and slimes) can overcome barriers to gene therapy by finely modulating the expression of transgenic vectors. These limitations include preventing genotoxicity due to gene delivery, modulating the nonspecific localization of the genome, increasing gene transfer efficiency, and reducing immune responses [177].

### Nonviral vector hydrogels for HCC

Nonviral vectors are less cytotoxic, immunogenic, and mutagenic than their viral counterparts [178] and have been explored for gene therapy in a variety of liver diseases, such as liver fibrosis, viral hepatitis, and HCC [179]. The ability to target specific genes makes polymeric nanoparticles, which can target cancer-specific DNA, particularly valuable [180]. These nanoparticles have shown promising antitumor effects both in laboratory settings and in animal models [181]. For example, cationic solid lipid nanoparticles effectively curtailed HCC growth by delivering short hairpin RNA (shRNA) targeting the NURP gene [182], and chitosan nanoparticles carrying small interfering RNA (siRNA) against the PLK1 gene also significantly inhibited HCC cell growth [183]. To achieve their potential, nonviral vectors require certain capabilities, including safeguarding genetic material against endonuclease degradation, ensuring efficient transport to and uptake by the nucleus, and facilitating vector unpacking. Tailored systems are often necessary to meet these requirements [178]. Hydrogels exhibit promise as delivery systems for nonviral vectors. Their unique physicochemical properties can maintain RNA bioactivity, enable sustained release, and provide localized delivery (e.g., via injection) of high concentrations of therapeutic agents to specific sites in an on-demand or pulsed manner through stimulus-responsive strategies [167]. RNA can be incorporated into hydrogels either directly or encapsulated in nanocarriers, thereby increasing the delivery efficiency, concentration, and targeting of RNA therapeutics (Fig. 6C to F). In summary, the adaptability of hydrogels supports their utility across various applications in regenerative medicine and their potential for nonviral gene delivery. The controlled administration of nonviral vectors via hydrogels is an emerging research area with great promise for the safe and effective treatment of diverse human diseases. This method has the potential to address current limitations in nonviral gene therapy, offering customizable platforms tailored to specific diseases [184].

## Biologic Hydrogels for HCC

Compared to conventional small-molecule drugs, biologics, whose peptide backbones range from small peptides to large mAbs (approximately 500 residues or 150 kDa), present enhanced safety, target specificity, and pharmacokinetic profiles [177]. Biologics have shown exceptional therapeutic potential in treating HCC, particularly for addressing the complex TME. By accurately modulating the activities of key immune cells in the TME, these agents can effectively amplify the body’s natural immune response to tumors [185]. Furthermore, these biological agents can target specific surface markers on tumor cells or crucial signaling molecules within the TME, allowing targeted cancer cell eradication while sparing normal cells [186,187]. However, the administration of biologics is commonly hindered by their low oral bioavailability and significant degradation within the GI tract, which necessitate invasive injection methods [177]. Moreover, the therapeutic dose range for biological drugs is extremely wide, spanning from nanograms/kilogram (agonists) to milligrams/kilogram (antagonists) [177]. Identifying patient-friendly administration routes and determining dosing flexibility are 2 significant breakthroughs that can be achieved through hydrogel delivery strategies and technologies. Hydrogels can serve as multifunctional drug delivery platforms that are adaptable to various administration routes, such as transdermal delivery or topical injection, and drug loading and release rates can be controlled by their physical properties, such as pore size and degree of crosslinking [12]. Hydrogels, as a customizable platform, offer a unique solution to the challenges of delivering biologics. The following section describes the combination of hydrogels with antibody and peptide drug therapeutics.

### Antibody–drug hydrogels for HCC

**Fig. 7.**
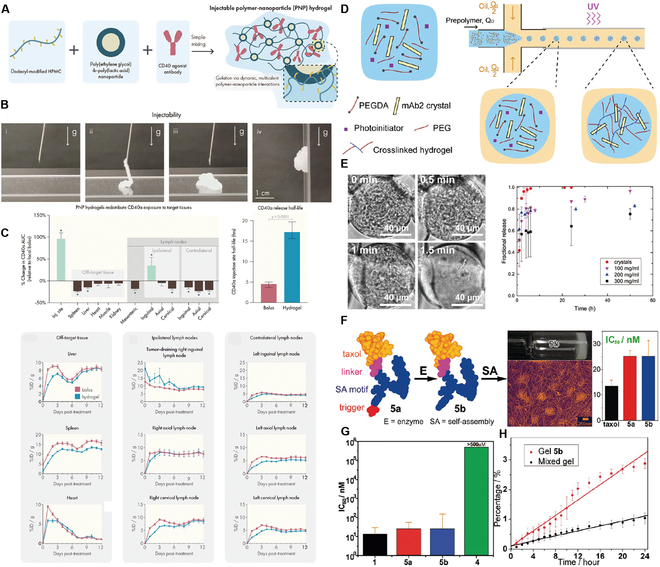
Hydrogel-assisted tumor biologics. (A) Injectable supramolecular polymer-nanoparticle (PNP) hydrogels composed of dodecyl-modified hydroxypropyl methylcellulose and poly(ethylene glycol)-b-poly(lactic acid) nanoparticles can be used to encapsulate CD40 agonists for local drug delivery. (B) PNP hydrogels exhibit solid-like rheological properties with robust shear-thinning and self-healing capabilities. (C) Sustained locoregional hydrogel-based delivery of CD40a slows tumor growth and improves safety. Reproduced with permission [190]. Copyright 2022 Wiley-VCH. (D) Schematic of the preparation strategy for hydrogel/crystalline microspheres; not to scale. (E) In vitro release of a hydrogel loaded with mAb2 crystals. Reproduced with permission [193]. Copyright 2021 Wiley-VCH. (F) By covalently linking PTX to easily self-assembled patterns, precursors (5a), hydrogelators (5b), and hydrogels of PTX derivatives were successfully generated without affecting the cytotoxicity of PTX. (G) Cytotoxicity of PTX (1), 5a, 5b, and 4 after 48 h of culture with HeLa cells (the *y* axis represents the log_10_ value). (H) Cumulative drug release profiles of 2 PTX gels in 100 mM phosphate-buffered saline (PBS) buffer. Reproduced with permission [206]. Copyright 2009 American Chemical Society.

Over the past 3 decades, therapeutic antibodies have significantly advanced targeted cancer therapy [188]. mAbs target specific antigens, inducing cytotoxic effects through neutralization or proapoptotic mechanisms and activating innate immune responses [189]. Despite their efficacy, the reliance on immunoglobulin G (IgG) antibodies for current antibody drugs presents challenges, such as limited penetration into solid tumors and the potential for Fc-mediated bystander immune system activation [186]. Hydrogels have emerged as an innovative solution to these challenges, enabling antibody miniaturization and the development of multifunctional approaches. For example, CD40 agonist antibody (CD40a) therapy, which both enlarges the anticancer T cell pool and modifies the TME to support these T cells, faces concerns about safety and tolerability. One study utilized an injectable supramolecular PNP hydrogel (Fig. 7A) composed of dodecyl-modified hydroxypropylmethylcellulose and poly(ethylene glycol)-b-poly(lactic acid) nanoparticles to significantly enhance the pharmacokinetics of CD40a therapy. This hydrogel allows localized, sustained release (Fig. 7B), increasing drug concentrations in the TME and tumor-draining lymph nodes (TdLNs), thereby promoting immune stimulation, drug safety, and anticancer effectiveness (Fig. 7C) [190]. Moreover, hydrogel microsphere formulations offer a faster, more patient-friendly administration route for mAbs, featuring high loading capacities and suitable flow properties for injection through narrow syringe needles with moderate force [191,192]. Concentrated suspensions of mAb crystals (>300 mg ml/l) can be encapsulated within hydrogel microspheres as small as 30 μm in diameter (Fig. 7D) [193]. Compared to the liquid and suspended crystal forms of mAbs, hydrogel formulations exhibit reduced viscosity and shear-thinning behavior, significantly increasing loading efficiency, stable crystallinity, and controlled release profiles of encapsulated mAb2 crystals while maintaining low viscosity at high loadings (Fig. 7E). Furthermore, hydrogels have been used to sustainably release antibody drugs while maintaining effective concentrations in the body [194]. A hydrogel made from sodium alginate–chitosan, for instance, was employed to regulate the release of IgG model antibodies and Fab antibody fragments. In summary, hydrogels not only concentrate drugs within the TME and prolong their retention but also increase drug safety and tolerability. Thus, hydrogels represent a promising, patient-friendly strategy for HCC treatment, highlighting the role of hydrogels in improving the delivery and therapeutic impact of antibody-based treatments.

### Peptide hydrogels for HCC

Peptides offer several advantages over antibodies, including superior tissue penetration, effective cell internalization, reduced immunogenicity, lower bone marrow and liver toxicity, and flexibility for chemical modifications [195–197]. They exert antitumor activity through a variety of mechanisms, such as disrupting cell membranes, inducing apoptosis, inhibiting tumor angiogenesis, modulating the immune response, and targeting specific intracellular pathways [198]. A growing number of peptide drugs have been the subject of recent research and development with the specific aim of providing a new treatment for HCC [199–201]. Despite these advantages, peptides face challenges such as their inability to cross cell membranes to reach intracellular targets and their vulnerability to hydrolysis or enzymatic degradation due to their amide bonds, resulting in compromised stability in vivo [202]. These peptides can also interact with nontarget cells, potentially leading to adverse reactions or cytotoxic effects [202]. Thus, increasing peptide bioavailability or stability through advanced administration methods or formulations is essential for their successful clinical use [203]. The use of hydrogel materials has emerged as a promising strategy in this regard. Wang et al. [204] created a pH-switchable nanofiber hydrogel network using cyclic phosphate and proline, which reacts to the acidic conditions of tumor sites to release peptides and drugs. Peptide–drug conjugates (PDCs) are a novel class of prodrugs that link specific peptide sequences to drugs via a cleavable linker, allowing the incorporation of multiple functional components within PDCs [205]. For example, Gao et al. [206] synthesized a supramolecular hydrogel for PTX delivery using a phosphorylated naphthalene-Phe-Phe-Lys-Tyr precursor conjugated to PTX. In the presence of a particular enzyme, this precursor transforms into a hydrogelator, self-assembling into nanofibers to form a supramolecular hydrogel that slowly releases the PTX derivative into aqueous medium for chemotherapy, preserving the cytotoxic effect of PTX (Fig. 7F to H). Although peptide drugs face specific hurdles in treating liver cancer, leveraging innovative drug delivery systems such as hydrogels can provide pathways to more effective and safer therapeutic options for patients. These advances highlight the potential of hydrogels to enhance the delivery and efficacy of peptide-based therapies in cancer treatment.

## Radiation Therapy Hydrogels for HCC

**Fig. 8.**
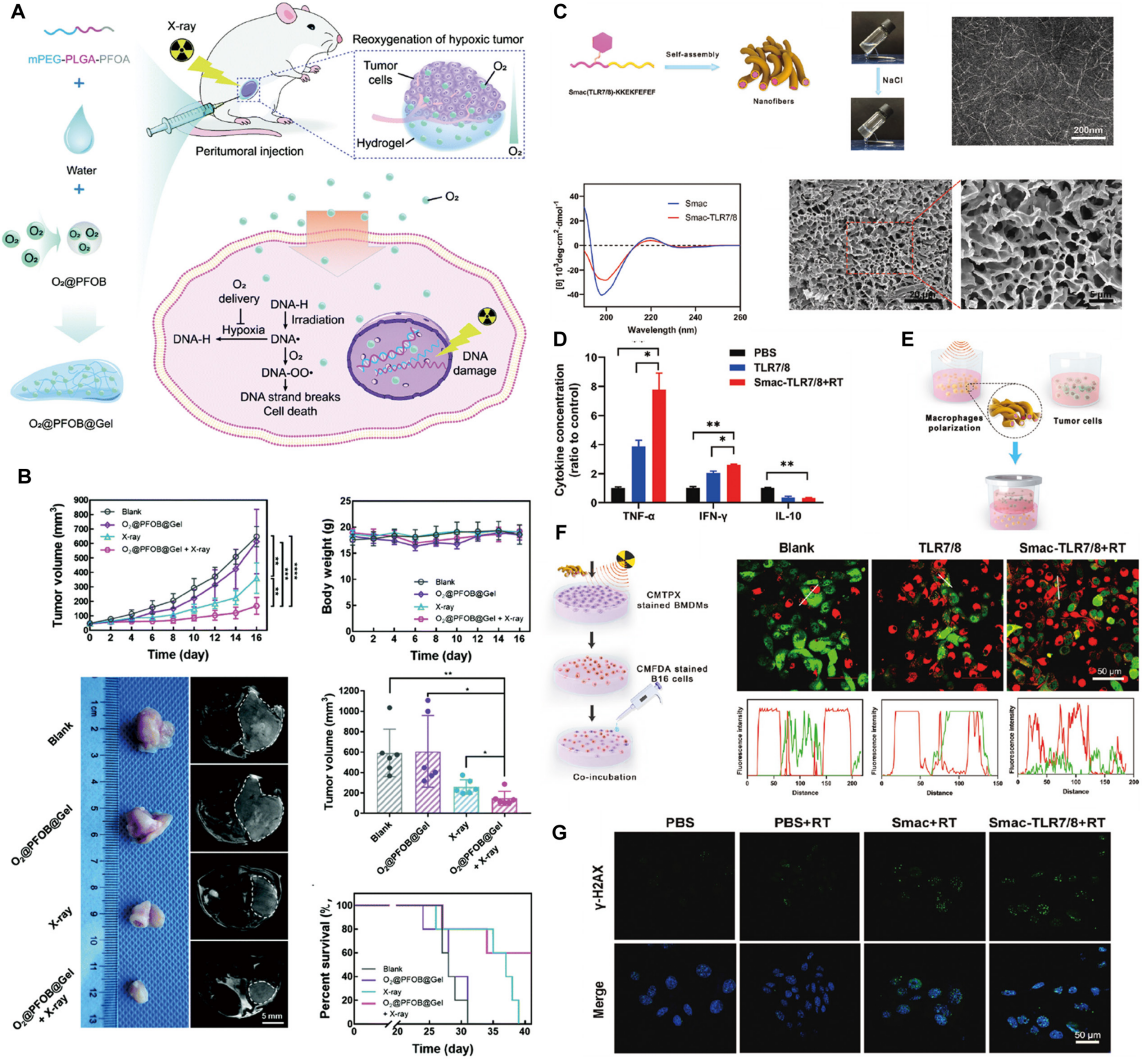
Hydrogel delivery strategy for RT. (A) Schematic representation of the preparation of an oxygen-enriched thermosensitive composite hydrogel (O_2_@PFOB@Gel) and its radiosensitizing effect on tumor radiotherapy. (B) Evaluation of the effects of an oxygen-enriched thermosensitive composite hydrogel in combination with radiotherapy on 4T1 breast tumor progression and patient survival. Reproduced with permission [217]. Copyright 2021 The Royal Society of Chemistry. (C) Characterization of Smac-TLR7/8 hydrogels. (D) Enzyme-linked immunosorbent assay (ELISA) for the levels of cytokines such as tumor necrosis factor-α (TNF-α), interferon-γ (IFN-γ), and IL-10 in the cell culture supernatant of bone marrow-derived macrophages (BMDMs) (*n* = 3). (E) Flow chart of the Transwell coculture cell system. (F) Schematic diagram of the assay of BMDM phagocytosis after treatment with different preparations and representative confocal images of the assay. Scale bar, 20 μm. B16 cells were stained with CMFDA (5-chloromethylfluorescein diacetate, green), and BMDMs were stained with CMTPX (CellTracker Red CMTPX). (G) Immunofluorescence imaging of intracellular γ-H2AX foci in B16 cells after treatment with PBS, Smac hydrogel, or Smac-TLR7/8 hydrogel under 6-Gy radiation. Scale bar, 50 μm. Reproduced with permission [220]. Copyright 2021 Elsevier BV.

Radiation therapy (RT) is a potent modality for treating unresectable HCC, achieving a 2-year local control rate between 60% and 100% [207,208]. It has become a standard treatment option and is especially beneficial for patients with portal vein thrombosis [209]. RT utilizes high-energy x-rays or gamma rays to inflict DNA damage or instigate the generation of large quantities of ROS [210]. When the pace of ROS production exceeds the cellular ability to detoxify these free radicals, cell death occurs. Additionally, radiation can induce immune cell death, promote immune response [211], and reprogram the TME [212], leading to autophagy in tumor cells. However, RT destroys tumor cells at the expense of adjacent cells and tissues [213,214], and high-dose radiotherapy has been linked to the induction of resistance. Conventional radiosensitizers such as mitomycin and 5-fluorouracil are ineffective in treating HCC [215], necessitating the development of highly selective and controllable radiosensitizers. The combination of hydrogel drug delivery systems and RT represents a novel approach to liver cancer treatment. By precisely releasing drugs, this strategy enhances the targeting and increase the safety of RT, thereby increasing the efficacy of low-dose radiation [216]. Yang et al. [217] developed an mPEG-PLGA-PFOA/PFOB composite solution, an oxygen-enriched thermosensitive hydrogel designed to supply exogenous oxygen steadily, reoxygenate hypoxic tumors, and counteract hypoxia-associated radioresistance, effectively inhibiting tumor growth (Fig. 8A). The PFOB-containing polymer solution exhibited satisfactory stability, remaining homogeneous after 3 d of storage at 10 °C. Animal studies revealed that the injection of O_2_@PFOB@Gel and subsequent oxygen release did not directly harm tumors, and stable body weight profiles indicated the absence of systemic toxicity of the treatments. Tumor volume was precisely measured through 3D magnetic resonance imaging (MRI) reconstruction, which revealed that the volume in the O_2_@PFOB@Gel+X-ray group was significantly smaller than that in the other groups (less than 200 mm^3^), highlighting the effectiveness of this approach (Fig. 8B). The intensive radiotherapy regimen led to a significant increase in the overall survival of mice treated with O_2_@PFOB@Gel+X-rays compared to that of the other groups. Despite this success, overcoming radioresistance remains a critical challenge in increasing the overall efficacy of radiotherapy treatments [218]. The causes of radioresistance are complex and are intimately associated with the TME [219]. The rapid proliferation and hypoxia of the TME leads to a chronic inflammatory environment around the tumor, gradually giving rise to an ITM [220]. After radiation, the secretion of inflammatory cytokines and the recruitment of immune cells may initiate immune attack on tumors but also increase radioresistance, immunosuppressive cell populations, and the levels of growth factors within the TME, thus complicating the immune response [221]. Conversely, radiosensitizing hydrogels can increase RT efficacy by reprogramming macrophage polarization to reconstruct the ITM and overcome radioresistance. Zhang et al. [220] introduced a TLR7/8-conjugated radiosensitizing peptide hydrogel (Fig. 8C). This hydrogel counteracts the radiotherapy-induced up-regulation of inhibitor of apoptosis proteins (IAPs) in tumor cells, which blocks apoptosis and reduces tumor radiosensitivity. The Smac peptide in the hydrogels directly binds to IAPs, inhibiting their function and enhancing tumor radiosensitivity [222]. Additionally, Smac activates TLR7/8-like receptors, promoting the polarization of macrophages to an M1 phenotype to remodel the ITM (Fig. 8D to G) [223,224]. In essence, the integration of RT with hydrogel drug delivery and the strategic use of radiosensitized hydrogels offer substantial benefits in improving radiotherapy outcomes for HCC patients. This approach not only enhances the treatment precision and safety but also addresses the issue of radioresistance, providing new avenues for liver cancer therapy and promising treatment outcomes for patients.

## TACE Hydrogels for HCC

**Fig. 9.**
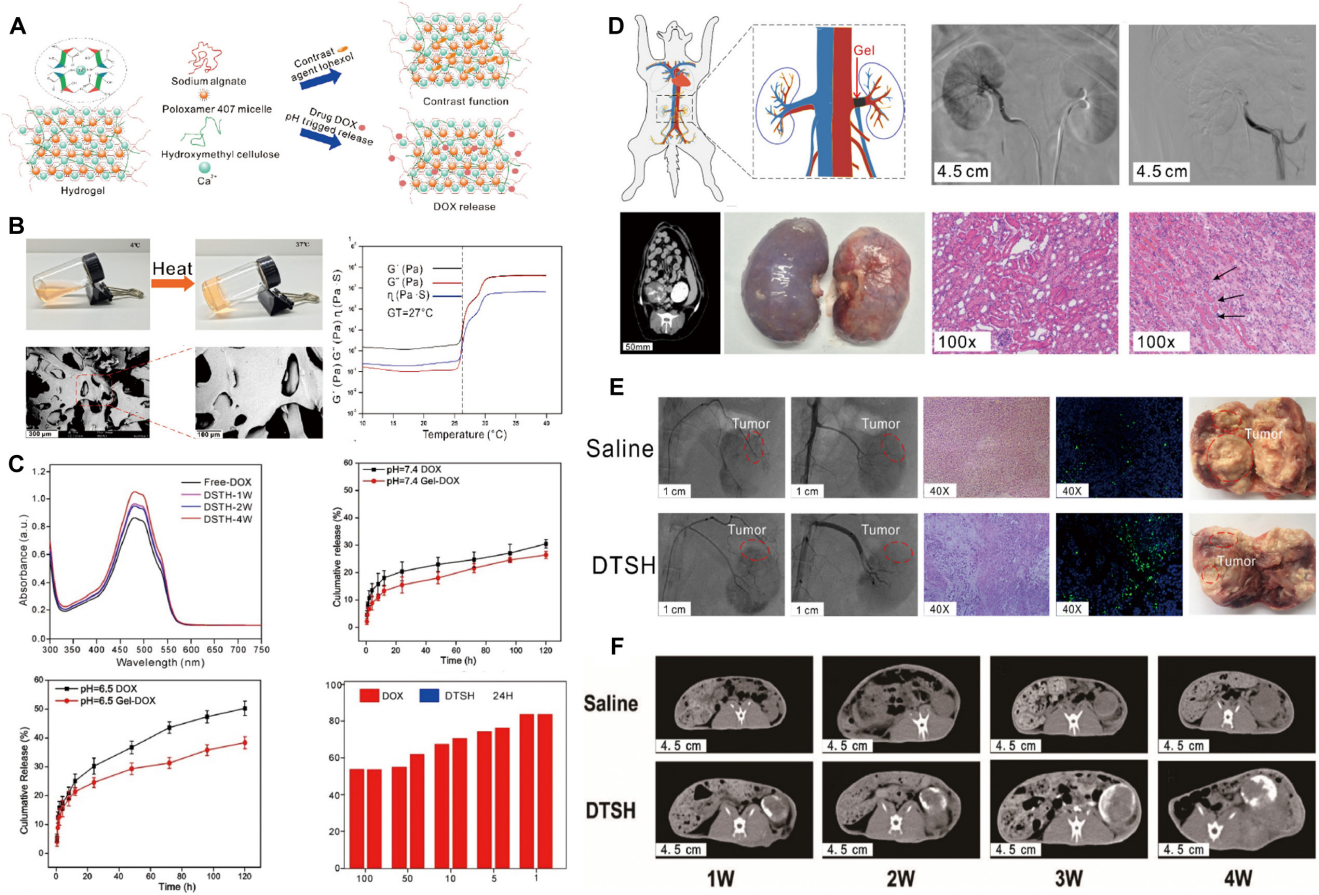
Hydrogel delivery strategy for TACE. (A) Preparation of the temperature-sensitive hydrogel. This hydrogel can be loaded with the anticancer drug DOX or the contrast agent iodohexol. (B) Thermosensitive sol–gel transition and rheological analysis and scanning electron microscopy characterization of DTSH. (C) Stability, doxorubicin release kinetics, cytotoxicity, and gelation characteristics of DTSH hydrogels under different pH conditions. (D) Evaluation of normal Beagle kidneys after renal artery embolization. (E) Comparative analysis of rabbit VX2 kidney tumors before and after treatment: angiographic images, histological staining, and apoptosis detection. (F) Computed tomography scan images of rabbits using saline and DTSH at intervals after embolization. Reproduced with permission [232]. Copyright 2021 Elsevier BV.

TACE is a targeted chemotherapy approach administered through a catheter that is inserted percutaneously and navigated under imaging guidance to the hepatic artery near the liver tumor for localized drug delivery. Currently, there are 2 modes: conventional TACE (cTACE) and drug-eluting particulate TACE (DEM-TACE). The key processes linked to the success of TACE are drug release and bulk transport through the vascular barrier and throughout the complex, variable TME [225]. In DEM-TACE, DOX is encapsulated within microspheres that gradually release the drug. However, these microspheres are unable to traverse the peribiliary capillary plexus into the tumor, leading to drug release in the distal arterial vasculature and subsequent diffusion or convection-mediated distribution [226,227]. Hydrogel microspheres, such as DCBead, HepaSphere, LifePearl, Tandem, DCB, and HepaSphere, benefiting from global approval and backed by extensive experimental and clinical research, are utilized as drug delivery systems for DEM-TACE [225]. These systems encapsulate DOX before the TACE procedure, offering controlled drug release to minimize systemic exposure to anthracyclines and associated side effects. For example, DCBead, made from crosslinked PVA hydrogel [228,229], enables sustained and localized drug delivery in a dose-dependent manner [230], achieving low systemic concentrations and high intratumoral levels of DOX to induce tumor necrosis and increase drug–cell interaction time [231]. Additionally, He et al. [232] developed temperature-sensitive hydrogels (DTSHs) loaded with adriamycin (Fig. 9A and B), which solidify at body temperature, ensuring the stable release of the drug within the TME (Fig. 9C). The application of DTSH in VX2 renal tumor treatment demonstrated effective embolic performance (Fig. 9D to F). Furthermore, the radiopacity of the hydrogel enables physicians to monitor the embolization process in real time through digital subtraction angiography (DSA). In conclusion, advances in TACE have provided novel opportunities for minimally invasive therapy, advanced the combination of embolization materials, including hydrogels, with radiographic techniques, and exhibited significant potential for precise drug delivery in complex TMEs.

## Ultrasonotherapy Hydrogels for HCC

**Fig. 10.**
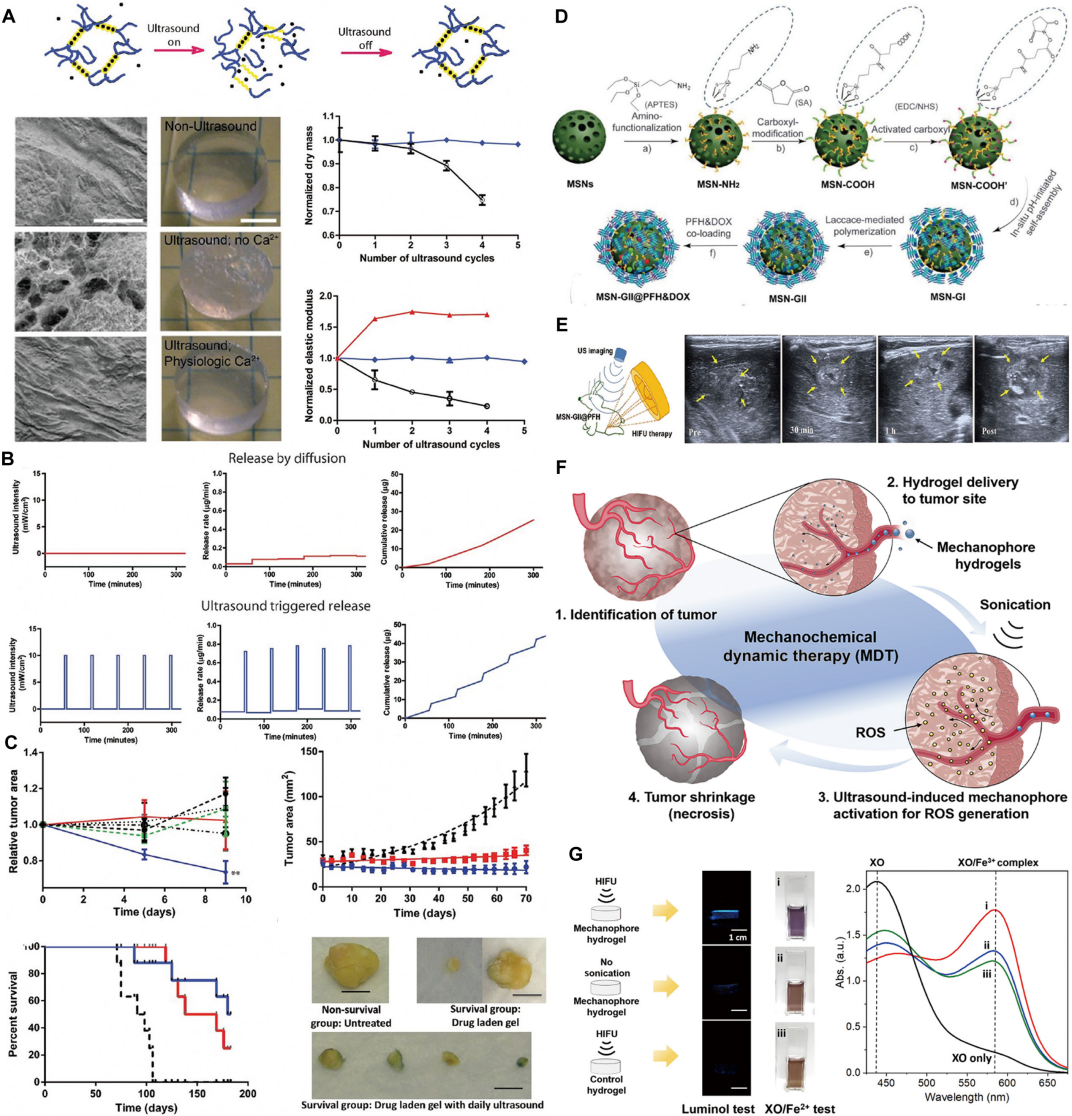
Hydrogel delivery strategy for ultrasound. (A) Ultrasound-induced disruption and self-repair of ionically crosslinked hydrogels. (B) Quantitative analysis of mitoxantrone release after different fusion (top) or sonication treatments (bottom; 5 min, hourly). The data show the ultrasound intensity (left), mitoxantrone release rate (center), and cumulative mitoxantrone release (right). (C) Effect of ultrasound-enhanced mitoxantrone ALG gel on tumor growth inhibition and survival. Reproduced with permission [236]. Copyright 2014 Proceedings of the National Academy of Sciences. (D) Schematic diagram of the ternary inorganic–supramolecular–polymer hydrogel system (MSN-GII@PFH&DOX). (E) Schematic of ultrasound imaging-guided HIFU treatment (left) and ultrasound image (right). Reproduced with permission [238]. Copyright 2019 American Chemical Society. (F) MDT concept, focusing on ultrasound-controlled mechanochemical reaction-generated ROS for noninvasive cancer therapy. (G) Luminescent phenol chemiluminescence and XO/Fe^2+^ colorimetric tests on a sonicated mechanohydrophobic gel (top), an unsonicated mechanohydrophobic gel (middle), and a sonicated control hydrogel (bottom). Reproduced with permission [239]. Copyright 2022 Proceedings of the National Academy of Sciences.

Ultrasound is a highly utilized modality in the medical field for its capacity for deep tissue penetration and focusing capability, particularly in HCC treatment, where its precision in focusing and controlling the temperature of a specific area can induce drug release in the target area. This effect is linked to elevated temperature, cavitation, and polymer degradation [233,234], and it can amplify drug uptake by tumor cells through acoustic diffusion effects [235]. Ultrasound can damage tissue; however, this destruction can be reversed with the use of self-healing hydrogels such as ALG, enabling on-demand drug delivery. Huebsch et al. [236] demonstrated that ionically crosslinked hydrogels can be disrupted by ultrasound through a dynamic mechanism. The cavitation effect of ultrasound disrupts the ionic cross-linking network between the metal ions and carboxylate groups, leading to a localized rupture and drug release. After the ultrasound stops, the dynamic ionic bonds reform, allowing the hydrogel to self-heal and re-establish its drug-encapsulating structure. Following the cessation of the ultrasound stimulus, these hydrogels can self-heal (Fig. 10A). This hydrogel was shown to be particularly effective at releasing the chemotherapeutic drug mitoxantrone (Fig. 10B), and the use of ultrasound-triggered bursts of high-dose mitoxantrone increased its toxicity to cancer cells compared to that achieved by continuous exposure (Fig. 10C). High-intensity focused ultrasound ablation (HIFU) is also an important treatment modality for HCC. The focused ultrasound field is precisely directed at deep target tissues through intact skin. Within seconds, the targeted tissue is heated to a temperature exceeding the threshold for protein denaturation. Specified tissue volumes can be thermally obliterated, while the skin and tissue layers outside the ablation zone remain relatively unaffected by ultrasound penetration [237]. Hydrogel nanoparticles impregnated with ultrasound-sensitive guest molecules can intensify the ablative effect of HIFU on tumors. Wang et al. [238] developed a ternary nanogel adsorbent, MSN-GII@PFH&DOX (Fig. 10D), using mesoporous silica nanoparticles (MSNs) as a multifunctional agent for both ultrasound imaging and imaging-guided high-intensity focused ultrasound (HIFU) therapy. Due to the elastic properties of the hydrogel shell, this ternary hybridized nanogel exhibited heightened contrast in ultrasound imaging. Additionally, ablation experiments have demonstrated a notable ability to increase the efficacy of HIFU treatments (Fig. 10E). The application of ultrasound was further expanded with the incorporation of azomechanical molecules in biocompatible PEG hydrogels, and Kim et al. [239] developed mechanochemical dynamic therapy (MDT) (Fig. 10F and G) as a cancer treatment platform, capable of the targeted release of ROS. Furthermore, the development of ultrasound-responsive composite hydrogels has broadened the application of ultrasound in liver cancer treatment, revealing novel therapeutic possibilities. The ultrasound-triggered response observed in hydrogels shows multifaceted capabilities that can enhance ultrasound imaging contrast, support image-guided drug delivery, and enable the on-demand pulse release of therapeutic agents [240]. The periodic mechanical stimulation provided by ultrasound-responsive microbubbles is instrumental in the field of stimulus response engineering, offering promising avenues for the development of cyclic therapy approaches for the treatment of liver cancer [11]. Ultrasound-responsive nanocomposite hydrogels are designed to undergo conformational changes and activate enzymes, facilitated by covalent intermolecular crosslinking. Ultrasound acts as a trigger to release drugs from within the crosslinked hydrogel, which in turn activates the enzymatic activities of molecules, such as transaminases [11]. Meng et al. [241] introduced an ultrasound-responsive self-healing hydrogel system that combines ultrasound with catalytic chemistry utilizing tetrabarium titanate electrodeposited nanoparticles. This innovative system achieves the targeted eradication of tumor cells by generating catalytic ROS under ultrasound irradiation, aiming for the remote control of tumor vaccine release and the customization of cancer immunotherapy. In summary, the synergy between ultrasound and hydrogels in liver cancer therapy exemplifies innovative integration. By integrating the precise focusing and temperature control capabilities of ultrasound with the highly efficient drug delivery potential of ultrasound-responsive hydrogels, tumor cells can be precisely targeted, and potential tissue damage can be substantially mitigated. This approach offers a safer and more efficacious method for HCC and simultaneously introduces new avenues for personalization and treatment monitoring.

## Ablation Hydrogels for HCC

**Fig. 11.**
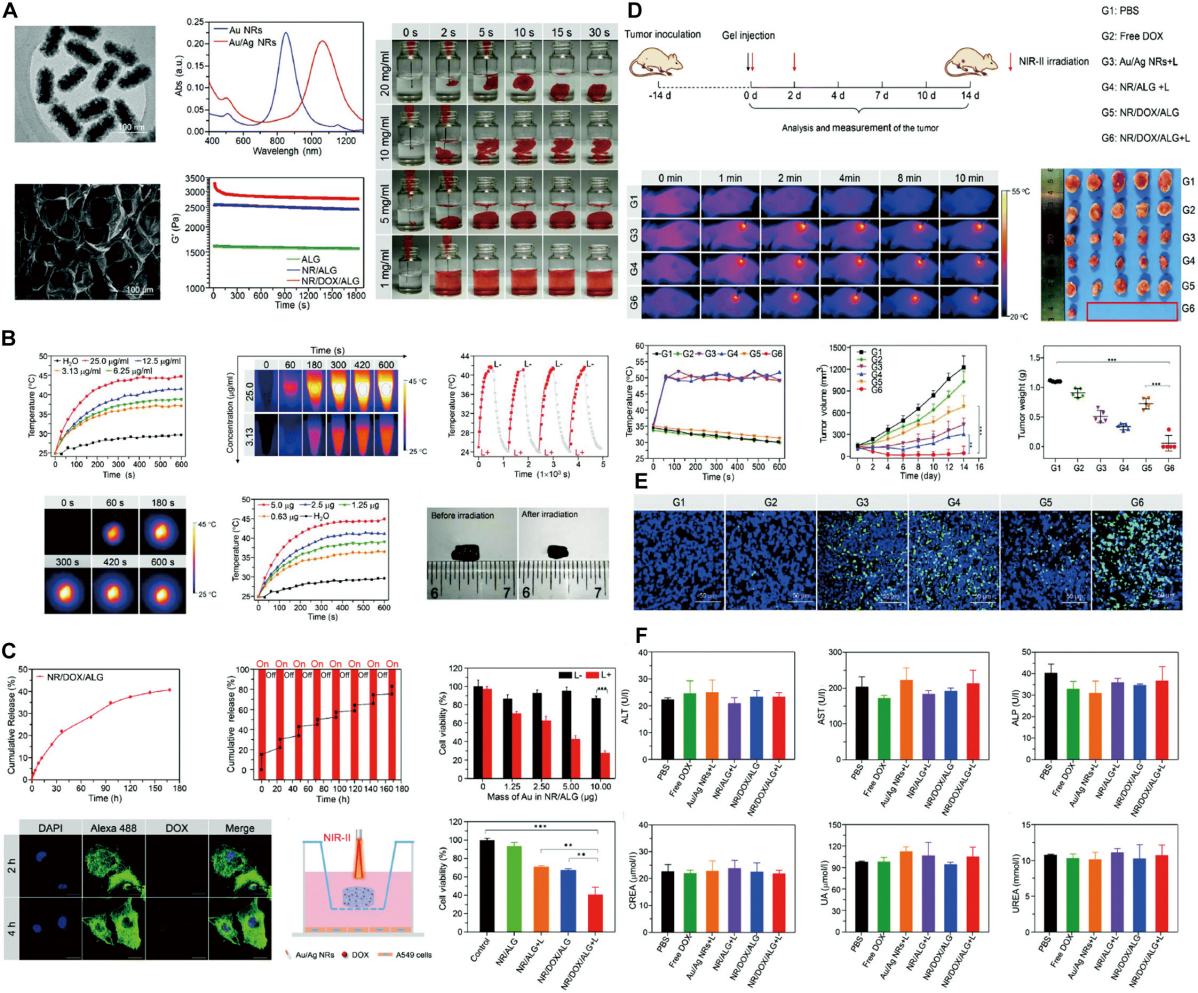
Hydrogel delivery strategy for ablation. (A) Preparation and characterization of the NR/DOX/ALG hydrogels. (B) Analysis of the photothermal effect of Au/Ag NRs under NIR-II laser irradiation and its application to drug release systems. (C) DOX release and combined anticancer activity triggered by the NR/DOX/ALG hydrogel. (D) Synergistic anticancer activity of the NR/DOX/ALG hydrogel. (E) Pathological changes in terminal deoxynucleotidyl transferase–mediated deoxyuridine triphosphate nick end labeling (TUNEL)-stained tumor tissues on day 14 after treatment (scale bar, 50 μm). (F) Comprehensive biochemical analyses revealed the effects of different treatment regimens on the indicators of liver and kidney function in the peripheral blood of mice: changes in the levels of alanine aminotransferase (ALT), aspartate transaminase (AST), alkaline phosphatase (ALP), creatinine (CREA), uric acid (UA), and urea. Reproduced with permission [249]. Copyright 2021 The Royal Society of Chemistry.

Ablation technology has become a pivotal approach in tumor treatment due to its significant benefits, including minimal invasiveness, providing rapid results, precision, minimal pain, and high efficiency [242,243]. Among the various ablation techniques, radiofrequency ablation (RFA) has traditionally been the most widely used method for treating HCC. However, microwave ablation (MWA) has gained popularity for early-stage HCC treatment in recent years, surpassing RFA. This shift is attributed to the ability of MWA to achieve higher ablation temperatures, shorten intervention times, and effectively counteract the heat sink effect, which can impair the efficacy of RFA [244]. Other liver ablation techniques, such as cryoablation and irreversible electroporation, remain under investigation [3]. However, the localized high temperatures produced by thermal ablation can harm adjacent tissues, leading to various complications [245,246]. Hydrogel can serve as an insulator to segregate normal tissue from the tumor ablation area, effectively mitigating adjacent tissue damage [247]. Huang et al. [248] developed a self-healing dynamic chitosan-PEG (CP) hydrogel that can be injected into the isolation zone, where it conforms to irregular tissue cavities, thereby creating a complete hydrogel shield to safeguard neighboring tissues. Hydrogels have the potential to act as a bridge integrating thermal ablation with pharmacological therapy. Wang et al. [249] developed an innovative injectable sodium ALG hydrogel that encapsulates corona gold/silver nanorods (NRs) together with the hydrophilic chemotherapeutic agent DOX hydrochloride (DOX-HCl) (NR/DOX/ALG hydrogel), enabling the combination of thermal ablation and chemotherapy for targeted cancer treatment (Fig. 11A). Upon local injection into tumor tissue, ALG quickly transforms into a hydrogel via Ca^2+^-mediated crosslinking, effectively localizing the Au/Ag NR photothermite and DOX-HCl within the tumor site. When exposed to 1,064-nm laser irradiation, the hydrogel exhibits a pronounced photothermal effect, leading to the ablation of the majority of tumor cells. The temperature increase induced by the hydrogel is dependent on its concentration, and slight shrinkage of the hydrogel samples due to water loss during photothermal activation can be observed, suggesting that the hydrogel has the capacity for controlled in situ thermal ablation (Fig. 11B). DOX, which is immobilized within the hydrogel, acts on residual tumor cells following thermal ablation, enhancing the treatment efficacy (Fig. 11C). The heat generated by the Au/Ag NRs also triggers the on-demand release of DOX from the hydrogel, optimizing drug delivery directly to the tumor site [249]. This innovative approach, which combines ablation technology with hydrogel delivery, not only achieves precise tumor ablation but also minimizes thermal damage to adjacent healthy tissues because the hydrogel functions as a protective barrier (Fig. 11D to F). Furthermore, the integration of thermal ablation with chemotherapy via hydrogels results in a potent synergistic effect, significantly improving therapeutic outcomes while reducing systemic side effects. This strategy introduces a novel, safer, and more effective method for the comprehensive treatment of HCC.

## Phototherapy Hydrogels for HCC

**Fig. 12.**
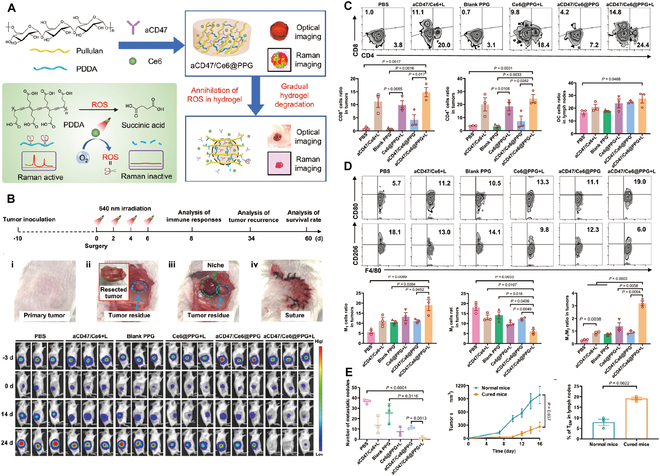
Hydrogel delivery strategy for phototherapy. (A) Schematic illustration of the construction of the CD47/Ce6@PPG hydrogel and traceable Raman ROS response degradation. (B) Experimental design and results of tumor resection and photodynamic therapy in a mouse model. (C) Impact of photodynamic therapy mediated by CD47/Ce6@PPG hydrogel on the immune response of tumor-infiltrating lymphocytes: Analysis of CD3^+^ T cell differentiation and the dynamic changes in mature dendritic cells (DCs) via flow cytometry. (D) Regulation of macrophage polarization and cytokine response in the tumor microenvironment under photodynamic irradiation with aCD47/Ce6@PPG hydrogel: Flow cytometry analysis reveals the dynamic balance between M1- and M2-type TAMs and the changes in key cytokine levels. (E) Metastasis prevention and long-term immunization effects of the photoirradiated aCD47/Ce6@PPG hydrogel in 4T1 hormonal BALB/c mice. Reproduced with permission [253]. Copyright 2022 Springer Nature.

Photodynamic therapy (PDT) operates by inducing the production of ROS, which are toxic to cells, thereby reducing cancer progression. This is achieved by causing microcirculatory dysfunction, which hinders the transport of essential nutrients and substances necessary for the survival, growth, and metastasis of cancer cells through the neovasculature surrounding the tumor [250,251]. Irreversible photodamage swiftly induces apoptosis and occludes neovascularization, thereby triggering necrosis and culminating in cancer death. However, there are multiple barriers to the clinical application of conventional PSs (photosensitizers), including low solubility, excitation by short-wavelength light, and photobleaching [252]. Combining ICB therapy with PDT has emerged as a promising strategy to improve tumor treatment by stimulating the immune system. However, a significant challenge arises when PSs and ICB antibodies are codelivered within a sealed carrier. The ROS produced during PDT within the carrier can destroy unreleased antibodies, significantly diminishing the effectiveness of the combined therapy [253]. Hydrogels present an innovative solution as sustainable codelivery systems for PSs and ICB antibodies that can effectively preserve antibody activity by neutralizing the detrimental ROS. Zhang et al. [253] created a biocompatible, ROS-responsive hydrogel, PPG, through the crosslinking of the conjugated polymer poly(deca-4,6-diynediynedioic acid) (PDDA) with the natural polysaccharide pullulan. This design exploits the full degradation of PDDA by chlorin e6 (Ce6) under red light irradiation to generate aCD47/Ce6@PPG (Fig. 12A). This hydrogel system simultaneously releases integrin-related protein antibody (aCD47) and Ce6. The result is a durable synergistic effect of PDT and ICB therapy that not only directly targets tumor cells but also sensitizes low-immunogenicity tumors and amplifies the therapeutic impact of aCD47. This effect was demonstrated by a decrease in bioluminescent signals and significant inhibition of recurrent tumor growth in treated mice (Fig. 12B to D), as well as the prevention of metastasis and induction of long-term immunization in these subjects (Fig. 12E). Additionally, the light sensitivity of light-responsive hydrogels can be leveraged to enable a noninvasive, precise, and remotely controllable activation mechanism [254], demonstrating their unique capacity for precise localization and controlled drug release in HCC treatment. Functional molecules can be integrated into hydrogels, and photocaged groups can be released via photochemical addition or photoisomerization reactions, while functionality can be eliminated through photochemical cleavage or photoaddition reactions [255]. Researchers have endeavored to bind PSs to the backbone of gel polymers for increased photothermal conversion and gel–sol transformation under near-infrared irradiation. For instance, poly(*N*-phenylglycine) (PPG), synthesized through the chemical oxidation of photoactive *N*-phenylglycine, was further modified with methoxy-PEG-amine via amide condensation to create a PEGylated PPG polymer (PPG-peg). This modification provided PPG-peg with a photothermal polymer backbone and facilitated the creation of a thermosensitized PPR (polymer–polymer recognition) through the established host–guest binding between PEG and α-cyclodextrin (α-CD). This interaction led to the formation of a photothermal network hydrogel, termed photothermal network hydrogel (PNT gel), via PPR interactions [256]. The PNT gel undergoes matrix disruption under the shear force of a syringe, transforming into a low-viscosity injectable fluid. Pulsed laser irradiation and the resulting light-triggered temperature rise are then utilized to release DOX from the PNT gel, producing a favorable anticancer effect. In summary, light-responsive hydrogels exhibit significant potential for liver cancer treatment, not only facilitating precise remote-controlled drug release but also enabling the integration of the long-term synergistic effects of PDT and ICB treatments to directly eradicate tumor cells and bolster the body’s immune response. Light-responsive hydrogels, characterized by their unique photothermal conversion capability and injectability, therefore significantly broaden the therapeutic possibilities for liver cancer treatment. This innovative approach not only increases treatment efficiency and safety but also introduces a level of flexibility and adaptability that was previously unattainable with conventional therapies. These characteristics provide new avenues for the future development of liver cancer treatments, promising more effective, minimally invasive, and patient-tailored therapeutic strategies.

## Electroactive Hydrogels for HCC

**Fig. 13.**
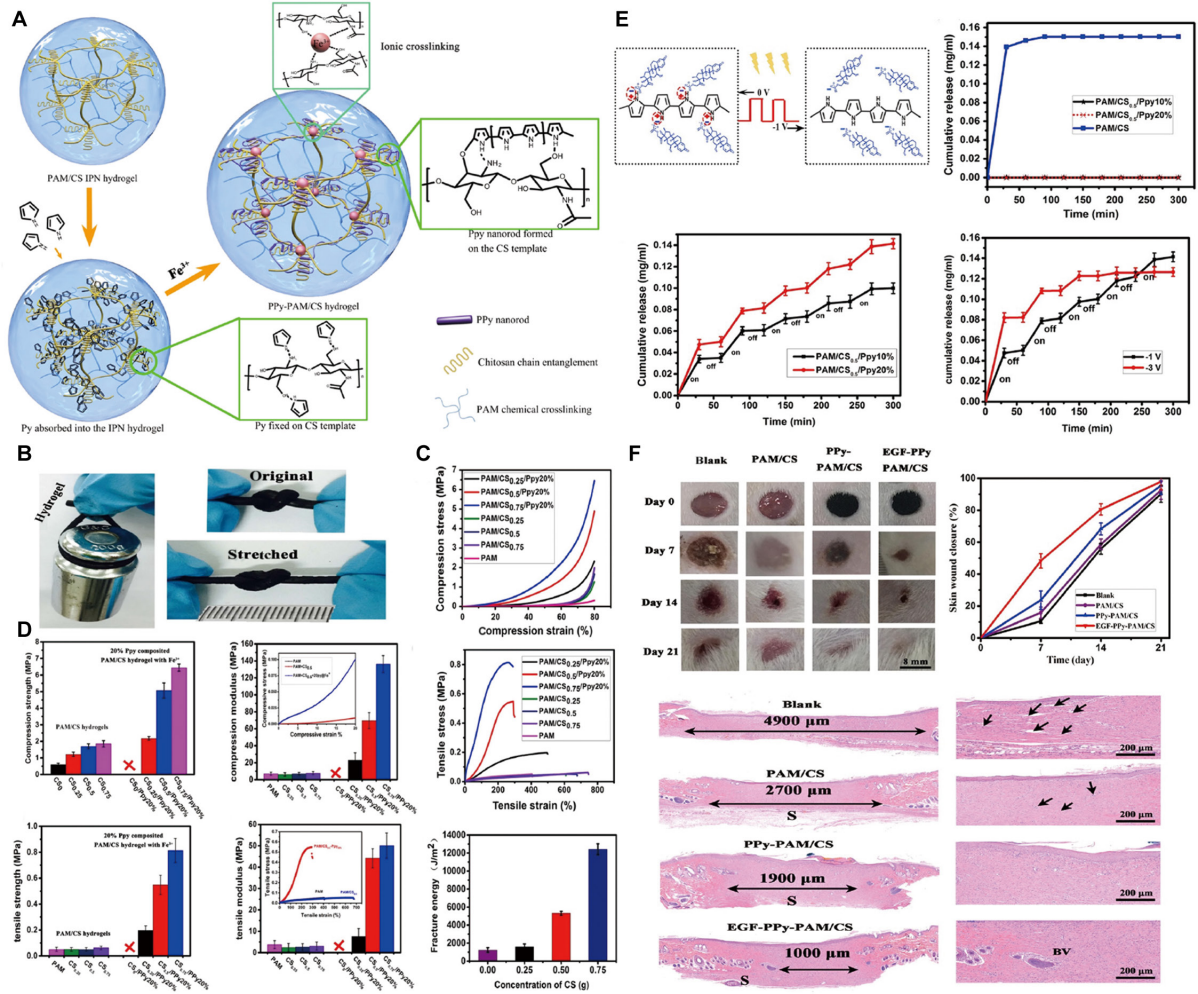
Hydrogel delivery strategy for electrochemotherapy. (A) Schematic of the PPy composite conductive and ductile hydrogel. (B) Photographs of PPy PAM/CS hydrogels (PPy 20 v/v%, CS 5 wt %) under loading or torsion. (C) Typical compressive stress–strain curves for various hydrogels. (D) Mechanical characterization of PAM/CS hydrogels with different CS concentrations and PPy compositions. (E) Schematic of dexamethasone (DEX) loading and release in hydrogels. The drug is released by a redox process when a negative potential is applied. (F) Evaluation of composite hydrogels in wound healing: time-series analysis of the effect of different formulations on defect healing and histological evaluation. Reproduced with permission [266]. Copyright 2018 American Chemical Society.

The term “electrochemotherapy” is used to describe the utilization of chemotherapy combining antitumor drugs with electric field pulses [257]. In this field, electroactive hydrogels based on conductive polymers are crucial in this field, enabling the precise spatiotemporal regulation of the release of various drugs through mechanisms such as actuation, charge transfer, and redox conversion upon electrical stimulation [258,259]. Electrode coatings derived from conductive polymers enable drug administration near the electrode implantation site, with significant implications for electrochemotherapy, and have been applied to cancerous tissues via electroporation following the injection of drugs such as bleomycin (BLM) or cisplatin [260]. Electrochemotherapy amplifies the cytotoxicity of drugs via electroporation, induces tumor vasoconstriction to minimize drug loss, obliterates the tumor vasculature, and activates an immune response, synergistically enhancing anticancer therapy efficacy [261]. Additionally, electroactive hydrogels are straightforward to prepare and notably robust [262]. For instance, Fantozzi et al. [263] coated electrodes with guar gum (GG) hydrogels swollen in BLM sulfate solution, facilitating electrochemotherapy. GG, a nonionic hydrogel, acts as a drug scaffold, remaining inert to electrical currents while permitting the migration of the ionic drug toward the counter electrode, and this movement can be directed to the tumor tissue via an electric field. Technological advances have led to the emergence of various electrically responsive hydrogels on the market. Hydrogels can be crafted from a variety of gel polymers notable for their electroactivity and processability, including polypyrrole (PPy), poly(3,4-ethylenedioxythiophene) (PEDOT), and polyaniline (PANI). Metallic nanoparticles such as gold, as well as carbon-based nanoparticles such as graphene and carbon nanotubes, are frequently integrated into hydrogel matrices to create electrically responsive hydrogels with expanded functionalities [264,265]. For instance, Gan et al. [266] engineered an electrically responsive interpenetrating hydrogel based on PPy NRs embedded within a chitosan/polyacrylamide (PAM) matrix (Fig. 13A). This hydrogel exhibits enhanced mechanical properties, electrical conductivity, and biocompatibility, marking a significant advance in the development of electrically responsive materials for various applications (Fig. 13B to D). The precise manipulation of electrical stimulation parameters, such as current intensity and duration, enables exact control of drug release within the liver tumor region (Fig. 13E). Moreover, in vivo implantation studies revealed that the polypyrrole-polyacrylamide/chitosan (PPy-PAM/CS) hydrogel effectively promoted skin wound repair (Fig. 13F). The integration of electrochemotherapy with electrically responsive hydrogels leverages the precision of electrochemotherapy alongside the dynamic modulation capabilities of electroactive hydrogels. This innovative combination allows the precise spatiotemporal control of drug release by electrical stimulation. This approach introduces a groundbreaking strategy for the treatment of HCC, joining electric fields with anticancer drugs to improve therapeutic targeting and efficiency. Moreover, this approach minimizes potential damage to healthy tissues through the protective and control functions offered by electroresponsive hydrogels. This methodology not only provides new avenues for HCC therapy but also underscores the potential of electroresponsive hydrogels in drug delivery systems, promising a more focused and less invasive treatment paradigm.

## Magnetotherapy Hydrogels for HCC

**Fig. 14.**
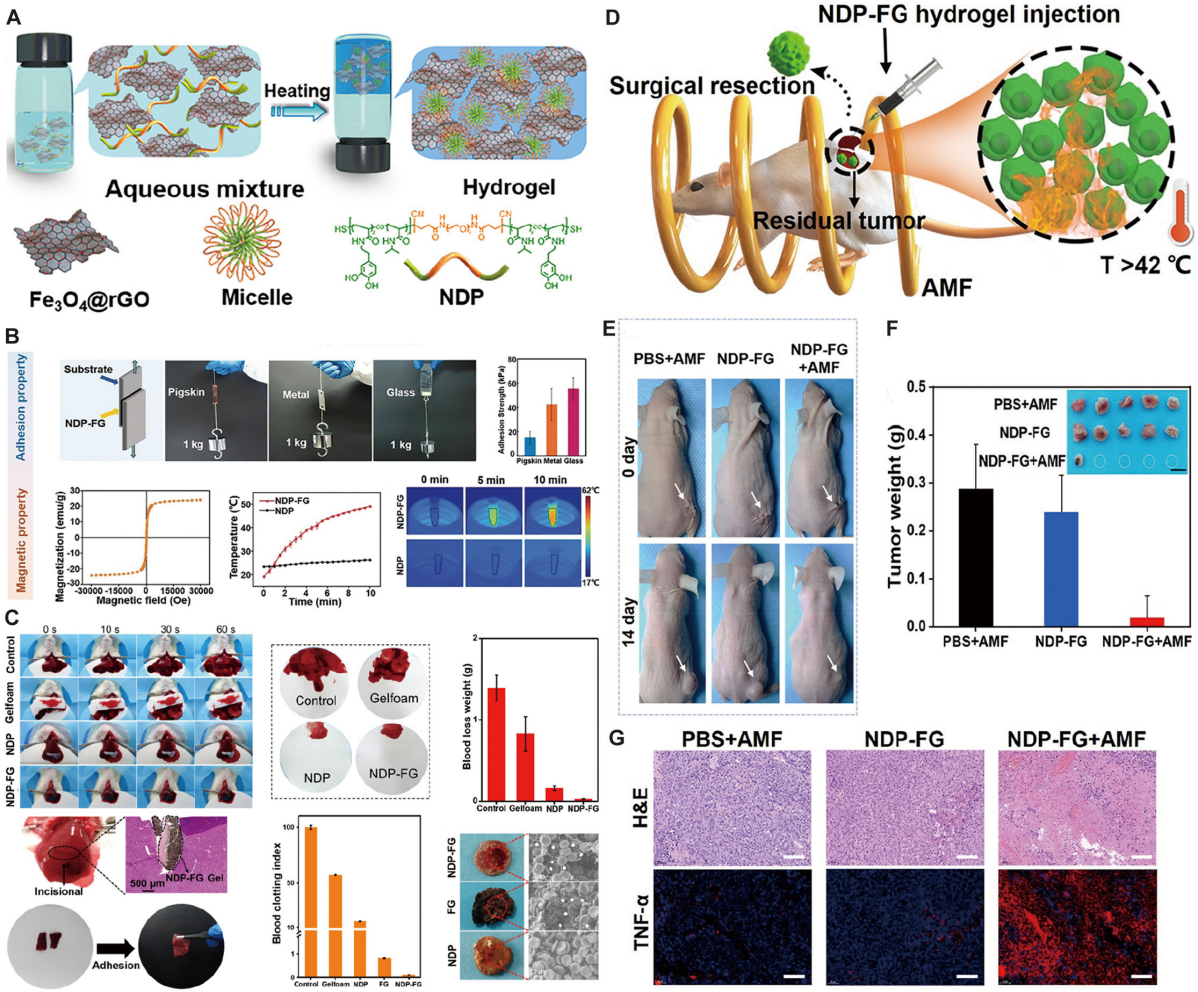
Hydrogel delivery strategy for magnetotherapy. (A) Schematic diagram of NDP-FG [poly(NIPAM-co-DOPA)-PEG-poly(NIPAM-co-DOPA), NDP; Fe_3_O_4_@rGO, FG] hydrodispersion during the sol–gel transition. (B) Characterization of NDP-FG hybrid hydrogels. (C) Hemostatic behavior of the NDP-FG hydrogel. (D) Schematic diagram of the induction of in vivo magnetic hyperthermia by the NDP-FG hydrogel to prevent cancer recurrence. (E) Photographs of mice in different treatment groups at 0 and 14 d after surgery. The arrows in the 0-d graph indicate wound closure, and the arrows in the 14-d graph indicate tumor recurrence. (F) Tumor weights of different groups at 14 d; inserted digital images are tumor tissues from different groups at 14 d. Scale bar, 1 cm. (G) H&E staining and TNF-α analysis of recurrent tumor sections from each group on day 14. Scale bar, 100 μm. Reproduced with permission [271]. Copyright 2018 American Chemical Society.

Magnetism is regarded as one of the optimal choices for the external stimulation of hydrogels due to its greater physical interaction with the body compared to other stimuli, such as light irradiation, ultrasound, or electric fields [267]. The incorporation of magnetic nanoparticles (MNPs) into hydrogel matrices results in magnetically responsive hydrogels whose physical properties can be remotely modulated under an external magnetic field. The ability to permanently alter their mechanical properties and porous structure enables precise spatiotemporal control of drug release [11]. The use of superparamagnetic particles, such as Fe_3_O_4_, γ-Fe_2_O_3_, and CoFe_2_O_4_, represents a cutting-edge method for treating liver cancer. These particles generate heat within the hydrogel through magnetic relaxation, which in turn triggers the controlled release of therapeutic agents [268–270]. A notable example is the body temperature-responsive in situ molded magnetic hydrogel reported by Yan et al. [271], which was constructed from reduced graphene oxide nanosheets (Fe_3_O_4_@rGO, denoted by FG) decorated with a triblock polymer matrix and iron oxide nanoparticles (Fig. 14A). Liver scratch studies and liver tumor resection experiments showed that this magnetic hydrogel exhibited robust adhesion and favorable injection properties under moist conditions (Fig. 14B) and exhibited hemostatic capabilities (Fig. 14C) and magnetic heat therapy (MHT) functionality (Fig. 14D to G) following hepatectomy. Furthermore, this hydrogel system can carry and release anticancer drugs, specifically PTX and DOX, through the magnetothermal effect present in magnetic supramolecular hydrogels (MSHs) [272]. MSH also has a pronounced thermokilling effect on cancer cells, suggesting potential for synergy with chemotherapeutic drugs for increased cytotoxic effects. Additionally, these superparamagnetic particles can facilitate MRI [273], enabling real-time monitoring and evaluation during liver cancer treatment. In summary, the advent of magnetically responsive hydrogels has led to multiple innovations in liver cancer treatment. From precise control of drug release to the integration of magnetothermal therapy and MRI, these strategies not only increase therapeutic efficacy but also offer tools for real-time monitoring to ensure precise and efficient treatment. Because of their great potential as intelligent drug delivery systems, magnetically responsive hydrogels provide an innovative and effective pathway for the future development of liver cancer treatment.

## Conclusions and Prospects

The treatment of HCC presents significant challenges due to the unique anatomical features of the liver and the intricate nature of the TME. The distinctive physiology of the liver offers a range of potential avenues for exploring treatment strategies for HCC. However, the TME plays significant roles in promoting drug resistance, facilitating tumor cell proliferation, and enabling immune escape. Collectively, these factors substantially hamper the effectiveness of traditional treatment modalities, such as surgery, local ablation, and chemotherapy. In light of these considerations, this paper presents a comprehensive overview of the multifaceted applications of hydrogels in liver cancer treatment, with a particular focus on combined use with specific therapeutic approaches for HCC and the role of responsive hydrogels in adapting to the complex microenvironmental properties of HCC, emphasizing their innovative and crucial role in HCC therapy. Hydrogels can facilitate precise drug delivery by encapsulating small-molecule chemotherapeutic agents and concentrating them in the TME, thereby increasing therapeutic efficacy and minimizing systemic toxicity. Concurrently, hydrogels can create a supportive microenvironment to enable immune cells to function effectively within the TME, mitigate tumor-induced immunosuppression, and facilitate the reactivation of the immune system’s capacity to target tumor cells. Moreover, hydrogels effectively safeguard sensitive therapeutic agents, including gene therapy drugs and other biologics, and ensure their targeted release in the TME. Furthermore, integrating hydrogel systems into therapeutic modalities such as RT, TACE, ultrasound therapy, ablation techniques, phototherapy, electrical stimulation, and magnetic therapy not only augments the efficacy of these treatments but also increases drug penetration into tumors, thereby improving the targeting and increasing the safety of drug delivery. In conclusion, the findings regarding the interaction of hydrogels with the TME in HCC and the properties of specific therapeutic strategies highlight the importance of hydrogels as efficient and versatile drug delivery platforms for HCC treatment. Consequently, further investigation into hydrogels in the context of HCC has the potential to not only facilitate significant advancements in the clinical application of tumor treatment but also offer novel insights and therapeutic strategies for future research.

While hydrogels have demonstrated considerable promise for clinical application as drug delivery systems and have attracted substantial research interest, their clinical translation presents several hurdles, including the following:

1.Technical challenges and storage stability in the construction of hydrogel delivery systems: The fabrication of hydrogel delivery systems involves complex technical processes, which can significantly limit their practical utility in clinical environments. A central issue is the potential risk of drug inactivation resulting from the instability or degradation of the drug before hydrogel network formation. Additionally, the storage stability of hydrogels poses a challenge for the clinical translation of hydrogel drug delivery systems. Drug delivery systems must maintain their performance and effectiveness under diverse storage conditions, including temperature, humidity, and light variations. To address this challenge, it is essential to select appropriate drug protection strategies, develop and implement stability management programs, and conduct regular quality control, monitoring of storage conditions, and stability testing to ensure that the product retains its performance during long-term storage.

2.Preparation homogeneity and batching for hydrogel clinical applications: Achieving a uniform distribution of drugs and active ingredients within the hydrogel matrix [274,275] poses a significant challenge in the fabrication of hydrogel delivery systems. This challenge not only impacts the consistency of drug release rates and therapeutic effects but also adds to the complexity of the preparation process. Additionally, performance differences or variations can exist among different batches of the preparation. These differences can arise from varying preparation conditions, raw materials, or other factors. Therefore, advanced preparation techniques and precise control strategies are essential to ensure the uniform fabrication of hydrogel drug delivery systems.

3.Challenges in clinical trials and approval: Hydrogel-based drug delivery systems encounter numerous challenges during clinical trials and approval processes. These challenges include achieving precise and sustained drug release in complex TMEs and meeting the rigorous standards of safety, efficacy, and precision mandated in clinical trials. Ensuring safety in clinical applications necessitates the comprehensive assessment of potential risks, the control of hydrogel concentrations, and an understanding of the potential effects of metabolites or degradation products on patients to minimize side effects and toxicity. Researchers must address the specific requirements of clinical trials and the complexities of regulatory approvals while enhancing and optimizing hydrogel drug delivery systems. The successful application and clinical approval of hydrogels for combination therapy will rely on meeting both technical and regulatory challenges, simultaneously ensuring both high safety and high efficacy.

**Fig. 15.**
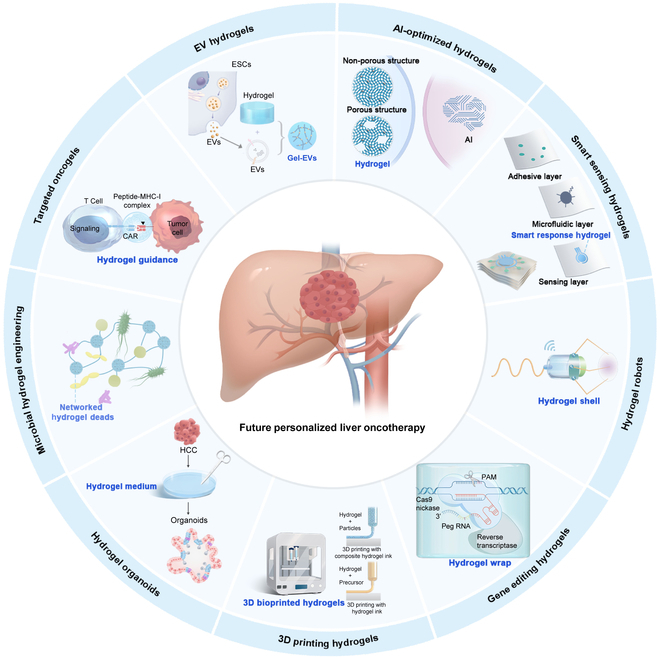
Future perspectives of hydrogels in the clinical management of HCC.

In conclusion, our findings underscore the significance of hydrogels as versatile drug delivery platforms for HCC treatment. By interacting with the TME and supporting specific therapeutic strategies, hydrogels hold great potential to advance clinical applications and provide novel insights for future research. To further enhance the therapeutic efficacy of hydrogels, it is crucial to address several technical challenges. The following 9 aspects (Fig. 15) represent key areas of focus for future hydrogel research, aimed at overcoming existing hurdles and facilitating the development of more precise and effective therapeutic strategies:

1.Artificial intelligence (AI)-optimized hydrogels: The integration of AI technology offers distinct advantages that set it apart as a transformative force in the field of hydrogel applications. AI provides an unprecedented ability to analyze complex datasets and model liver cancer-specific microenvironments, enabling the precise control of drug release from hydrogels. This allows for the design of more efficient and accurate drug delivery systems tailored to the unique characteristics of each patient. Moreover, AI facilitates the customization of hydrogel systems based on individual biomarkers, paving the way for truly personalized therapies that can adapt to the dynamic nature of liver cancer progression. The integration of AI into hydrogel research is expected to revolutionize the field by enhancing treatment efficacy, minimizing adverse effects, and ultimately improving patient outcomes. By harnessing AI’s computational power, researchers can optimize hydrogel formulations and predict therapeutic responses, positioning AI-optimized hydrogels as a next-generation solution for targeted cancer therapies.

2.Smart sensing hydrogels: Future research on sensing technology should include the integration of hydrogels with biosensors and conductive wearable or implantable biodevices to develop more advanced and multifunctional platforms. Such platforms have the potential to detect various liver cancer-related biomarkers, monitor drug metabolism, and assess therapeutic efficacy, enabling comprehensive monitoring of liver cancer treatments and providing physicians with instantaneous data on disease progression to facilitate the optimization of treatment strategies [276,277]. Furthermore, wireless smart hydrogel bandages represent an innovative therapeutic tool in postoperative liver cancer care. This system integrates sensors and stimulators to enable the active monitoring and closed-loop treatment of wounds via hydrogel electrodes that are closely integrated with the tissue interface. In conclusion, smart monitoring systems for the treatment of diseases such as liver cancer are anticipated to enhance therapeutic efficiency and contribute to the development and optimization of personalized treatment plans.

3.Hydrogel robots: Hydrogel robots demonstrate significant potential in liver cancer treatment. These miniature robots, constructed from highly flexible and responsive hydrogel materials, can navigate precisely to the targeted lesion for treatment [278]. When combined with microelectromechanical system (MEMS) technology, these robots can execute precise drug delivery, perform minimally invasive surgeries, and directly destroy tumor cells in the liver through mechanical means. Advances in nanotechnology and biomedical engineering have enabled the creation of hydrogel robots that can be programmed to react to specific biochemical signals [279], including molecular markers unique to liver cancer. This capability facilitates the highly efficient targeted treatment of liver cancer cells. The emergence of hydrogel robots was a significant advance in the field of liver cancer treatment, introducing more precise and minimally invasive alternatives. This development not only heralds a new era of innovation in treating liver cancer but also sets the stage for the future exploration of novel therapeutic strategies, potentially transforming the landscape of cancer treatment.

4.Gene-editing hydrogels: Hydrogels serve as effective carriers for delivering gene-editing tools, such as CRISPR–Cas9 [280], to liver cancer cells or tissues, thereby enabling innovations in cancer therapy. The excellent biocompatibility and modifiable degradation properties of these materials render them ideal media for gene delivery. This approach increases the precision and safety of gene editing in liver cancer therapy, reducing the risks of nonspecific editing and off-target effects. The use of gene-editing hydrogels provides novel possibilities for the precise localization and editing of liver cancer-related gene variants, facilitating the development of personalized and accurate treatments for liver cancer.

5.3D-bioprinted hydrogels: 3D bioprinting technology allows researchers to create fully personalized hydrogel systems that precisely match a patient’s anatomical features and therapeutic needs by meticulously controlling the hydrogel geometry, pore structure, and composition. Hydrogels exhibit significant potential as specialized tissue engineering scaffolds, particularly in the field of liver tissue engineering. They can facilitate the regeneration and repair of damaged liver tissues, providing an environment conducive to the growth and reformation of healthy liver cells [281]. Additionally, they can function as precision drug delivery carriers, directing drugs specifically to liver cancer cells or affected tissues. These applications not only improve treatment targeting and increase efficacy but also offer more innovative and personalized approaches for the surgical and localized treatment of liver cancer.

6.Hydrogel organoids: The exploration of hydrogel organoids in liver cancer research represents an innovative direction in this field [282]. In this technique, hydrogels are used to create miniature biological models that resemble the human liver, providing a realistic 3D environment for studying the biological properties and drug responses of liver cancer cells. Furthermore, these hydrogel organoid models hold significant potential for use in the high-throughput screening of small-molecule drugs targeting HCC, potentially accelerating the discovery and optimization of new drugs. By simulating the responses of liver cancer cells under simulated physiological conditions, this approach increases the efficiency and accuracy of drug screening, preparing the way for new possibilities in liver cancer treatment.

7.Hydrogels for microbial community modulation: Future research is expected to involve increasing focus on integrating hydrogels with microbial communities, particularly the application of conditionally pathogenic bacteria and beneficial anaerobes. Precisely manipulating these microbial populations using hydrogels will enable the elucidation of their roles in regulating liver metabolism, the immune system, and the TME. Particularly regarding gut microbiota regulation and the impact of the gut–liver axis, these innovations signal the emergence of new therapeutic avenues and will become vital tools for exploring host–microbe interactions, thereby providing expanded possibilities for HCC treatment.

8.Novel tumor-specific targeting hydrogels: The development of hydrogel systems capable of targeting novel molecular markers specific to liver cancer is an important direction in the advancement of future liver cancer therapies. Future hydrogels could be meticulously engineered to identify and adhere to specific targets associated with liver cancer cells. An example of such a target is the polymeric immunoglobulin receptor (pIgR), which is found in circulating extracellular vesicles (EVs) [283]. The discovery of this novel biomarker has introduced new avenues for research on liver cancer therapy. Future hydrogel systems could be custom-tailored to target these HCC markers, leading to highly precise drug delivery, optimized therapeutic efficacy, and reduced side effects. Additionally, these hydrogels could be employed for the early detection and monitoring of liver cancer progression, enabling treatment personalization and increased precision. This advance signifies substantial progress in liver cancer treatment toward more precise and efficient therapeutic strategies.

9.EV hydrogels: The incorporation of EVs into liver cancer therapy signifies a new chapter in hydrogel applications. EVs, which are key carriers in intercellular communication [284], are rich in biomolecules, including proteins, RNA, and DNA. The integration of EVs into hydrogels creates therapeutic systems that possess both targeting capabilities and bioactivity. Such systems, which combine EVs with hydrogels, are engineered to deliver therapeutic molecules directly to HCC cells with high precision. Additionally, they can effectively modulate signals within the TME, thereby increasing therapeutic efficacy against HCC. The integration of EVs and hydrogels represents a forward-thinking approach for the treatment, diagnosis, and monitoring of HCC. This combination not only has the potential to significantly improve therapeutic outcomes but also introduces new opportunities for innovative therapeutic strategies, offering promising prospects for advancing the management and understanding of HCC.

By integrating these technologies and introducing further innovation, future hydrogel systems can be made smarter, more effective, and more personalized, thereby revolutionizing the treatment of diseases such as liver cancer.
